# Merkel cell polyomavirus exploits extracellular vesicles for skin infection and host immune evasion through activated Wnt signaling

**DOI:** 10.1371/journal.ppat.1014360

**Published:** 2026-06-23

**Authors:** Alexander M. Pham, Luz E. Ortiz, Han Chen, Neil Christensen, Hyun Jin Kwun

**Affiliations:** 1 Department of Cell and Biological Systems, Penn State University College of Medicine, Hershey, Pennsylvania, United States of America; 2 Penn State Cancer Institute, Hershey, Pennsylvania, United States of America; 3 Transmission Electron Microscopy Core, Penn State College of Medicine, Hershey, Pennsylvania, United States of America; 4 Department of Pathology and Laboratory Medicine, Hershey, Pennsylvania, United States of America; 5 Penn State Clinical and Translational Science Institute, Hershey, Pennsylvania, United States of America; University of Wisconsin Madison School of Medicine and Public Health, UNITED STATES OF AMERICA

## Abstract

Research on small DNA tumor viruses has been limited by the lack of advanced virus culture systems that support robust viral infection. Thus, studies involving Merkel cell polyomavirus (MCPyV), a human oncogenic polyomavirus, primarily rely on model systems that might not fully reflect the native MCPyV skin infection. To decipher the mechanisms of natural human polyomavirus skin infection, we developed a 3D spheroid system for producing MCPyV virions derived from primary human dermal fibroblasts. The 3D spheroid microenvironment enhanced viral genome replication and genome maintenance, enabling the production and secretion of MCPyV virions that were associated with infectious extracellular vesicles (MCPyV-EVs) into the culture medium. EV-mediated MCPyV infection was moderately susceptible to antibody-mediated neutralization, implying the importance of EV-mediated MCPyV infection in host immune evasion. Proteomic analysis revealed a significant decrease in antiviral interferon-stimulated genes (ISGs) within MCPyV-EVs, indicating that MCPyV utilizes EVs as a means to enhance susceptibility of uninfected cells for viral transmission and infection. RNA-Seq gene expression analysis of primary human dermal fibroblasts with MCPyV infection suggests that the 3D microenvironment could replicate Wnt-mediated epithelial remodeling in the skin. Our results imply that Wnt-driven wound healing processes, accelerated by skin damage, UV radiation, and aging, regulate MCPyV viral replication and productive infection, which may promote the development of MCPyV-associated MCC.

## Author summary

To overcome the difficulties in studying MCPyV skin infection, a 3D virus culture system efficiently producing infectious virions using primary skin dermal fibroblasts was developed for the first time. Notably, MCPyV virions were found to be packaged and associated with extracellular vesicles (MCPyV-EVs), thereby uncovering alternative routes of infection and novel mechanisms of immune evasion for MCPyV. The 3D microenvironment resembled Wnt-mediated epithelial remodeling during natural skin wound healing processes, a microenvironment that could enhance virus entry and productive infection. MCPyV infection in 3D cell culture impaired the host antiviral immune system by downregulating the JAK/STAT and MAPK signaling pathways, which are critical for immune-mediated viral clearance. MCPyV-EVs cargo loading changes in ISGs, IFITM3, MIF, Galectins, and TGFBRs indicate a coordinated mechanism to attenuate antiviral and TGF-β signaling, driving immune suppression. Our findings provide significant insight into the mechanisms of MCPyV-associated skin cancer by understanding MCPyV infection and immune evasion strategies.

## Introduction

Human tumor viruses pose an imminent public health threat, as the rate of virus-induced cancer cases is steadily increasing [1,2]. Merkel cell polyomavirus (MCPyV) is a double-stranded DNA tumor virus that predominantly induces a persistent asymptomatic skin infection but can cause a highly aggressive skin cancer called Merkel cell carcinoma (MCC) due to a combination of additional factors, such as chronic exposure to UV radiation, immunosuppression, and aging [2,3]. Though MCPyV is associated with 80% of MCC cases, limited information is known about the viral lifecycle and its interactions with the host due to its poor ability to replicate in traditional cell culture [4]. To date, common approaches used to generate recombinant MCPyV virus-like particles (VLPs) or quasiviruses are often produced from HEK293T/TT cells and supplemented with exogenous MCPyV proteins to increase packaging efficiency of recombinant virions for infection studies [5–8]. Although infections with recombinant viruses have been used in discerning aspects of MCPyV tropism and lifecycle, these recombinant systems were neither robust nor naturally recapitulated the skin infection as serial transmission of the virus is difficult to detect [5–8].

Challenges in studying the mechanisms of human tumor virus oncogenesis and infection arise due to the lack of *in vivo* animal models, confining most studies to the traditional *in vitro* two-dimensional (2D) monolayer cell culture system [9,10]. Three-dimensional (3D) culture is used to mimic the tissue microenvironment *in vitro* and is more physiologically representative of the complex organization and interactions of cells in tissue and organs compared to 2D monolayer cell culture [11,12]. These intricate interactions within the 3D cellular environment are essential for cell differentiation, morphology, cell signaling, and subsequently critical for cell function [13,14]. As such, several studies have reported that the 3D structure of host cells promotes efficient viral replication, genome maintenance, and host permissibility of human tumor viruses. Notably, upregulation of the metabolic pathways in 3D spheroids has elucidated mechanisms of genome maintenance of Kaposi’s sarcoma-associated herpesvirus (KSHV) [15]. Long-term 3D culture of hepatitis B virus (HBV) fully recapitulated the viral lifecycle and innate immune response observed in HBV patients [16]. Furthermore, the complete lifecycle of human papillomavirus (HPV) and virus production was first elucidated using 3D organotypic raft cultures of epithelial tissue [17,18]. Similarly, efficient replication of Epstein-Barr virus (EBV) in stratified epithelium was detected *in vitro* [19]. Thus, 3D cell culture models have been beneficial for investigating aspects of the natural host microenvironment and could also help advance studies for other human tumor viruses, in which cell culture models are limited.

In this study, we cultured MCPyV in primary skin fibroblasts to determine whether the 3D microenvironment could support the MCPyV lifecycle and virion production. We found that the 3D microenvironment substantially enhanced MCPyV genome replication and maintenance, enabling the production of MCPyV virions that were packaged into extracellular vesicles (MCPyV-EV). MCPyV-EVs efficiently infected primary dermal fibroblasts and were moderately resistant to neutralizing antibodies. The augmented viral replication in 3D culture could be attributed to the endogenous activation of the Wnt-driven signaling pathway, and subsequent downstream matrix metalloprotease (MMP) expression and disruption of the actin cytoskeleton in fibroblasts. These processes are fundamental to Wnt-mediated epithelial remodeling during wound healing. During tissue repair, Wnt signaling is upregulated to guide cellular reorganization, migration, and differentiation. The 3D microenvironment accurately resembled the host gene expression during skin wound healing processes, demonstrating that the unique physiological properties within the skin permits human polyomavirus infection.

## Results

### Various scaffold-free 3D culture platforms support MCPyV replication and transcription

**Fig 1:**
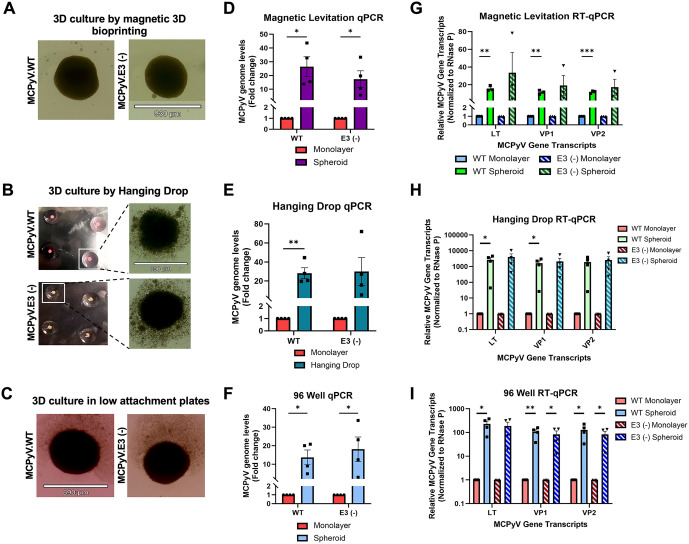
MCPyV genome replication and gene expression are enhanced in 3D spheroid cultures of primary human fibroblast cells. Three methods of 3D spheroid formation were used to culture primary neonatal human dermal fibroblast cells (nHDF) including magnetic levitation/bioprinting-based spheroid using gold and iron oxide nanoparticles (NanoShuttle-PL), hanging drop, and low attachment suspension culture. nHDF cells were transfected with the MCPyV wild type genomic clone (MCPyV.WT) or a mutant genomic clone of MCPyV with increased replication capacity (MCPyV.E3(-)) [7] and cultured as a 2D monolayer or 3D spheroid. Spherical aggregates of nHDF cells ranged from 500-600 µm in diameter with no distinct morphological differences. **(A)** Light microscope images of spheroids formed with magnetic bioprinting using NanoShuttle-Poly-L-Lysine. Scale bar = 930 µm. **(B)** Spheroids generated by a hanging drop method. Scale bar = 890 µm. **(C)** Spheroid suspension culture. MCPyV-transfected cells were grown as 3D spheroids cultured in 96-well U-bottom plates. Scale bar = 890 µm. **(D-F)** MCPyV genome replication is significantly increased in 3D compared to 2D culture. MCPyV viral genome levels in 2D and 3D cultures of nHDF cells were analyzed by qPCR at 4 days post-transfection (n = 4). MCPyV genome levels were 10-30-fold increased in 3D spheroid cultures. **(G-I)** MCPyV transcripts were increased in 3D compared to 2D culture. Viral transcripts isolated from 3D culture were determined by RT-qPCR and normalized to monolayer cultures at 4 days post-transfection (n = 4). RNAse P was used as a housekeeping gene for RT-qPCR analysis. Expression of both early (LT) and late gene (VP1 and VP2) transcription was substantially upregulated in 3D compared to 2D culture. Unpaired multiple Student’s *t* test using the Holm-Sidak method were used to test statistical significance and error bars are reported as standard error of the mean (SEM).

Primary human dermal fibroblasts (HDF) have been identified as a key, permissive host cell type that supports the complete MCPyV life cycle [5]. While these skin fibroblasts are susceptible to infection, they exhibit surprisingly low infectivity and require a significant amount of culture condition modulation to improve infection efficiency, which does not reflect the natural characteristics of the host environment. Thus, we asked whether 3D culture of primary HDF could be used to better model MCPyV skin cell infection. To generate 3D spheroids, primary neonatal human dermal fibroblasts (nHDF) cells were transfected with a minicircle genomic clone of the wild type (WT) or mutant (E3(-)) MCPyV [20,21]. The E3(-) mutant contains point mutations in serine residues (S220A/S239A) of the large tumor antigen (LT) which allows for enhanced replicative capabilities [7,22] (Fig 1). Since the wild type virus replication is highly restricted in a standard approach, we included the mutant virus as a positive control to ensure that experimental assays were sensitive enough to detect replication. Cells transfected with the MCPyV genome were cultured as 3D spheroids using three different methods and compared to traditional 2D monolayer cultures (Fig 1A–1C). To make scaffold-free 3D spheroids, we utilized NanoShuttle-PL, a mixture of iron oxide and gold nanoparticles that are attached to poly-L-lysine, to coat the cell surface and magnetize the cells [23,24]. Magnetized cells were then seeded into low attachment plates for spheroid bioprinting using magnetic levitation (Fig 1A). Other 3D spheroids were generated using hanging drop (Fig 1B) [25] and low attachment suspension plating methods (Fig 1C).

MCPyV-transfected nHDF cells cultured in all three scaffold-free spheroid methods had a 10–30-fold increase in MCPyV genome replication of both WT and E3(-) compared to 2D cultures (Fig 1D–1F). Early and late gene transcripts for MCPyV LT, VP1, and VP2 were also consistently upregulated in both WT and E3(-) in 3D cultures compared to 2D (Fig 1G–1I). Likewise, MCPyV transcripts were greatly elevated in 3D using other human fibroblast cell lines (primary adult human dermal fibroblast (aHDF) and BJ-hTERT cells) (S1 Fig in S1 Appendix). Taken together, our results demonstrate that 3D spheroid cell culture supports enhanced MCPyV genome replication and viral gene transcript expression compared to the traditional 2D monolayer culture.

### MCPyV genome is efficiently maintained in 3D spheroid cell culture

**Fig 2:**
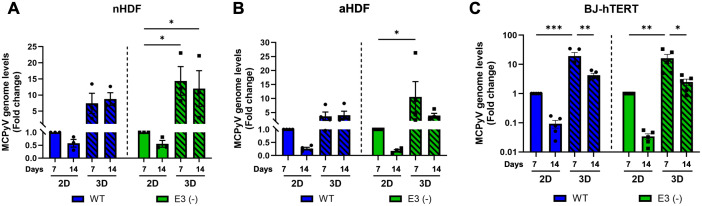
The 3D spheroid microenvironment substantially supports MCPyV viral genome maintenance in human skin fibroblasts. MCPyV genome maintenance was markedly increased in 3D cultures of **(A)** primary nHDF (n = 3), **(B)** primary adult human dermal fibroblasts (aHDF) (n = 4), **(C)** and BJ-hTERT cells (n = 5). MCPyV genome levels were measured by qPCR in 3D spheroids generated by magnetic levitation/bioprinting at 7- or 14-days post-spheroid formation. MCPyV genome levels were normalized to monolayer culture (2D) 7 days after seeding. MCPyV genome was efficiently maintained in 3D culture while viral DNA was rapidly lost in 2D culture of human skin fibroblasts. Statistical significance was determined using a two-way ANOVA with Tukey’s multiple-comparison tests and error bars represent standard error of the mean (SEM).

Given that 3D spheroid skin cell culture involves aggregating skin cells in a scaffold-free environment to mimic *in vivo* tissue architecture, we sought to address whether this 3D microenvironment could support MCPyV genome maintenance. To determine how MCPyV DNA levels changed over time in 2D or 3D cultures, cells with MCPyV genome were seeded as 2D monolayers or 3D spheroids formed via magnetic levitation and cultured for 7 or 14 days. More than 50–90% of the virus genome was lost over 7 days in 2D culture, while 3D spheroids maintained steady-state levels of MCPyV genome over time in nHDF cells (Fig 2A). The maintenance of MCPyV DNA in spheroids was phenocopied in both aHDF (Fig 2B) and BJ-hTERT cells (Fig 2C). Importantly, in all experiments and timepoints, MCPyV DNA was maintained at elevated levels in spheroids compared to monolayers, exemplifying the ability of 3D culture to efficiently support MCPyV replication and genome maintenance.

### 3D culture of MCPyV in primary skin fibroblasts enables MCPyV virion production and release into the culture supernatant

**Fig 3:**
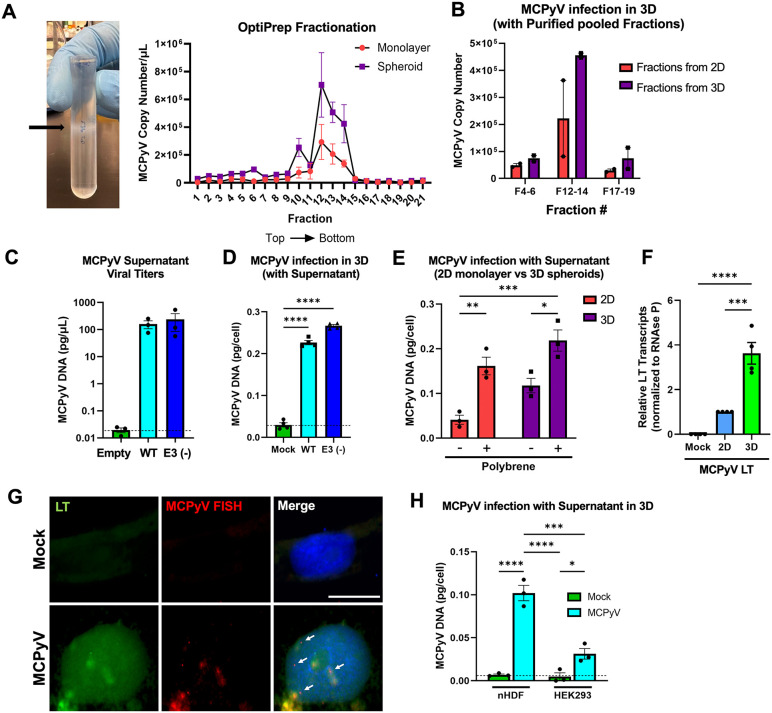
Three-dimensional culture of MCPyV enables the production and substantial release of infectious MCPyV virions into the supernatant. **(A)** OptiPrep density gradient ultracentrifugation and fractionation. nHDF cells were transfected with MCPyV and cultured in 2D or 3D for 4 days. MCPyV virions were isolated over a discontinuous 27-39% OptiPrep density gradient. MCPyV genome copy numbers were determined in each fraction. MCPyV virion production was consistently higher in 3D compared to 2D culture. Left: OptiPrep gradient with arrow indicating the band containing MCPyV virions. Right: Detection of MCPyV DNA in OptiPrep gradient fractions isolated from 2D or 3D culture (n = 3). **(B)** MCPyV infection with virions isolated from OptiPrep fractions. Pooled OptiPrep fractions were used to infect nHDF cells. Total genomic DNA was isolated and analyzed for MCPyV DNA detection (n = 2). Virus particles isolated by OptiPrep fractionation from 3D and 2D cultures efficiently infected nHDFs on a cell-repellent plate. **(C)** MCPyV virions are released from host cells. nHDF cells were transfected with an empty vector (mock) or the wild type (WT) or E3 ligase mutant (E3 (-)) MCPyV genome and cultured as 3D spheroids. Mock or viral supernatants isolated from transfected spheroids were examined for MCPyV DNA via qPCR at 4 days post-transfection (n = 3). MCPyV virions are produced and released into the supernatant. **(D)** MCPyV virions produced from spheroids are infectious. nHDF cultured as spheroids were infected with mock or viral supernatants (2 x 10^8^ MCPyV genome copies) and harvested 4 days post-infection (n = 4). Statistical significance was analyzed using a one-way analysis of variance (ANOVA) with Dunnett’s multiple-comparison tests. **(E)** MCPyV infection is more efficient in 3D. Spheroid or monolayer nHDF cultures were infected with viral supernatants (2 x 10^8^ MCPyV genome copies) supplemented with or without polybrene, a cationic polymer (12 μg/mL) for 4 days (n = 3). MCPyV infection efficiency is substantially augmented in the 3D microenvironment and could be further enhanced through polybrene treatment. Statistical significance was determined using a two-way ANOVA with Tukey’s multiple-comparisons test and error bars represent SEM. **(F)** MCPyV gene expression is enhanced in infected spheroids. RT-qPCR analysis of MCPyV LT transcripts from MCPyV-infected (2 x 10^9^ MCPyV genome copies) monolayers or spheroids (n = 4). RNAse P was used as a housekeeping gene. Statistical significance was analyzed using ANOVA with Dunnett’s multiple-comparison tests. **(G)** MCPyV DNA detection by FISH analysis. nHDF cells were infected with supernatants (2 x 10^8^ MCPyV genome copies) and probed for LT expression (green) and MCPyV DNA (red) at 4 days post-infection. Arrows denote positive MCPyV FISH signal. MCPyV DNA detected by fluorescent *in situ* hybridization (FISH) strongly colocalized with condensed nuclear LT. Scale bar = 70 μm. **(H)** Host-restricted infection of skin-derived MCPyV virions. HEK293 or nHDF spheroids were infected with mock or MCPyV supernatants (2 x 10^8^ MCPyV genome copies) for 4 days (n = 3). MCPyV virions derived from skin fibroblasts infect skin cells more efficiently than HEK293 cells. Statistical significance was determined using a two-way ANOVA with Tukey’s multiple-comparisons test and error bars represent SEM.

Since we observed that MCPyV genome replication was enhanced in the 3D culture of primary nHDF cells, we asked whether this 3D microenvironment allowed for the generation of infectious virions. To determine this, we first isolated MCPyV virions from cell lysates by OptiPrep gradient ultracentrifugation [8]. A band was observed in the middle of the gradient, which corresponded to fractions (F) 12–14 containing peak amounts of MCPyV DNA (Fig 3A). Each fraction from cell lysates of 2D and 3D cultures was compared for virus genome levels. Virus genome levels in 3D cultures were consistently higher (4–7 x 10^5^ genome copies/μL) in fraction numbers 12–14 compared to 2D (1–3 x 10^5^ genome copies/μL) (Fig 3A). To determine whether these fractions contained infectious MCPyV virions, we pooled OptiPrep fractions (F4-6, F12-14, F17-19) from either 2D or 3D cultures and infected nHDF cells on a cell-repellent plate. Notably, we could detect a high MCPyV infection of OptiPrep fractions F12-14 from both 3D spheroids and 2D monolayers (Fig 3B).

Next, we determined whether MCPyV virions were released into the supernatant from 2D and 3D cultures (Figs 3C and S2A in S1 Appendix). MCPyV genome was efficiently detected in the supernatant of 3D cultures (~100–300 pg/μL). Similar to the viral titers from the OptiPrep fractionation, the detection of MCPyV genome levels from 2D supernatants (~50–60 pg/μL) were significantly decreased compared to 3D supernatants. Importantly, the supernatants isolated from 3D cultured nHDFs with MCPyV were infectious when incubated with normal nHDF cells on a cell-repellent plate (Fig 3D), indicating the successful production of MCPyV virions from 3D culture. Of note, the infectivity of MCPyV supernatants derived from 3D culture was higher in spheroids (0.1-0.2 pg/cell) compared to standard 2D monolayers  (<0.02 pg/cell) (Fig 3E). The infection efficiency in both 2D monolayers and 3D spheroids could be enhanced by treating the viral supernatants with polybrene, as it neutralizes the electrostatic repulsion from the plasma membrane.

To further confirm the presence of infectious virions in the supernatants, RT-qPCR was conducted to observe MCPyV gene expression in infected monolayers and spheroids. MCPyV LT transcript levels were significantly upregulated in 3D-infected nHDF cells compared to 2D-infected cells (Fig 3F). Additionally, MCPyV DNA was detected in the virus-infected cells using fluorescent *in situ* hybridization (FISH). The MCPyV genome strongly colocalized with condensed nuclear LT expression, suggesting the formation of MCPyV replication centers (Fig 3G). Both MCPyV LT and VP1 capsid protein expression were also observed in the cells infected with MCPyV in 3D via immunofluorescence (S2B Fig in S1 Appendix). In addition, protein expression of MCPyV LT was observed from infected spheroids via immunoprecipitation coupled with immunoblot analysis (S2C Fig in S1 Appendix). To examine the cellular tropism of MCPyV virions isolated from skin cells, HEK293 cells, a cell type often used to generate recombinant MCPyV VLPs and quasiviruses [5,6,8], were infected with viral supernatants and infectivity was compared with nHDF cells (Fig 3H). Infectivity in HEK293 was substantially lower compared to nHDF cells, indicating that MCPyV virions derived from fibroblasts exhibit higher susceptibility to their specific natural reservoir cell types.

### MCPyV utilizes extracellular vesicles as an alternative route for skin infection

**Fig 4:**
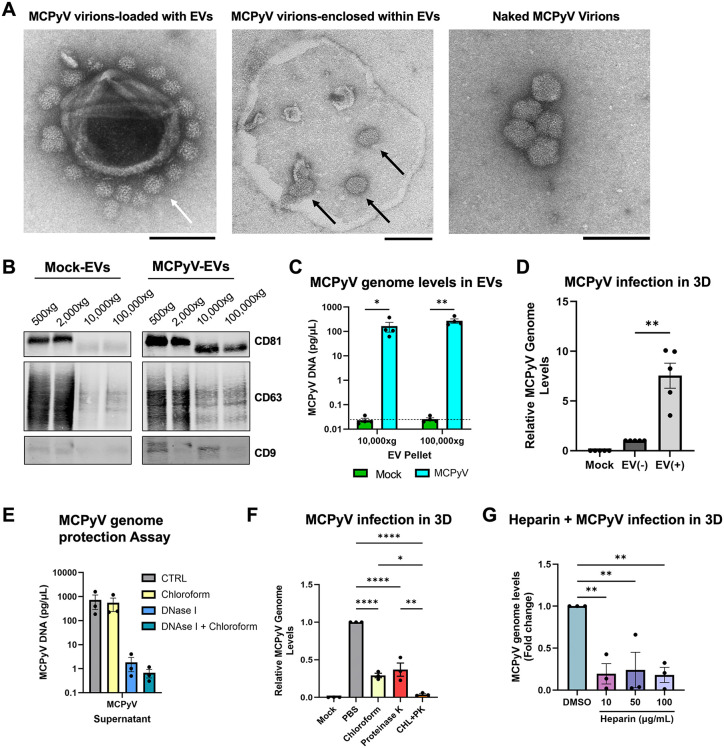
MCPyV utilizes extracellular vesicles for efficient infection in natural host cells. **(A)** MCPyV virions are associated with EVs. Extracellular vesicles (EVs) were isolated by differential centrifugation using the supernatants from nHDF cells transfected with MCPyV at 4 days post-transfection. Negative TEM staining of MCPyV-EVs purified from the 10,000 x g pellet. MCPyV virions are often loaded on the surface of EVs (left) or enclosed within EVs (middle). Naked EV-free virions are also observed (right). White arrow: MCPyV virions tethered to EVs. Black arrow: MCPyV virions found inside of EVs. Scale bar = 100 nm. **(B)** MCPyV-EVs are positive for EV markers. Pellets isolated by differential centrifugation were probed for the expression of tetraspanins (CD9, CD63, CD81), common EV markers (n = 3). Equal volumes of protein lysates were loaded for immunoblot analysis. **(C)** Secreted MCPyV virions are associated with EVs. EVs were isolated from nHDF spheroids transfected with the MCPyV genome by differential centrifugation. The 10,000 x g and 100,000 x g pellets were probed for the MCPyV genome via qPCR (n = 4). Statistical significance was determined using a two-way Analysis of Variance (ANOVA) using Fisher’s LSD multiple-comparison tests and error bars represent standard error of the mean (SEM). **(D)** MCPyV utilizes EVs for infection. nHDF cells were infected (2 x 10^9^ MCPyV genome copies) with viral supernatants enriched in EVs (EV(+)) or depleted of EVs (EV(-)) for 4 days. EV(+) viral stocks were made by resuspending the 100K EV pellet in PBS. EVs were depleted from supernatants through centrifugation at 100,000 x g for 2 hours (n = 5). MCPyV infection with EVs (EV(+)) is more efficient than infections with naked MCPyV virions alone (EV(-)). Unpaired two-tailed Student’s *t* test was used to test significance. **(E)** MCPyV genome protection assay. Viral supernatants were treated with 10% chloroform and/or DNAse I (120 units/mL) and then analyzed for MCPyV DNA via qPCR (n = 3). Supernatants were extracted with chloroform, which solubilizes lipids and aids in the purification of the naked virus, EV-free MCPyV virions. To degrade any free, non-encapsidated DNA in the supernatant, DNAse I was used. Viral DNA isolated after chloroform extraction followed by DNase I treatment comes only from protected, intact virus particles. **(F)** Virus infectivity after Chloroform and Proteinase K Treatment. Viral supernatants were incubated with chloroform (CHL, 1:1 by volume) or Proteinase K (PK, 50 μg/mL) to solubilize EV membranes or degrade naked virions respectively (n = 3). For dual treatments of CHL + PK, viral supernatants were first extracted with chloroform and then treated with PK. CHL + PK treatment releases EV-encapsulated MCPyV virions, enabling exposure to PK and resulting in the complete degradation of all naked virions. Treated supernatants were then used to infect (2 x 10^9^ MCPyV genome copies) nHDF spheroids for 4 days. CHL + PK completely abrogated MCPyV infection, confirming the packaging of MCPyV virus particles inside of EVs. Statistical significance was analyzed using ANOVA with Dunnett’s multiple-comparison tests. **(G)** Heparin blocks MCPyV infection. Cells were pretreated with heparin for one hour and then infected with MCPyV supernatants (2 x 10^8^ MCPyV genome copies) for one hour (n = 3). Heparin, a highly sulfated glycosaminoglycan, inhibits both MCPyV-EV uptake and naked MCPyV infection by blocking their attachment and entry into cells. Statistical significance was analyzed using ANOVA with Dunnett’s multiple-comparison tests.

To observe and verify the production and release of MCPyV virions into the culture supernatant, viral supernatants were collected and analyzed by negative staining transmission electron microscopy (TEM). Surprisingly, besides the observation of naked MCPyV virions, we found that virus particles were also associated with extracellular vesicles (EVs) (Fig 4A). MCPyV virions were observed to be completely encircled and tethered to the surface of EVs and were also found on the inside of ruptured EVs (Fig 4A). MCPyV virion size ranged from 35-50 nm in diameter (S3 Fig in S1 Appendix). To verify EV-associated MCPyV virion production (MCPyV-EVs), EVs were further isolated using differential centrifugation [26]. MCPyV-transfected spheroids and their associated cell culture supernatant were subjected to spins at 500 x g, 2,000 x g, 10,000 x g, and 100,000 x g to pellet cells, debris, large EVs, and small EVs, respectively. Purified EV pellets (10,000 x g and 100,000 x g) were positive for EV tetraspanin markers, CD9, CD63, and CD81 [27] (Fig 4B). When MCPyV-EV pellets were probed for viral DNA, EVs were highly enriched in MCPyV DNA (200–300 pg/μL) (Fig 4C). To verify if EVs are utilized for MCPyV infection, supernatants were depleted of EVs through centrifugation at 100,000 x g to produce EV-free media (EV(-)) containing only naked MCPyV virus particles. Cells were then infected with EV(-) or EV-enriched (EV(+)) media (2 x 10^9^ MCPyV copies) (Fig 4D). Cells infected with EV(+) media had an enhanced infection efficiency, ~ 7.5x greater than cells infected with EV(-) media, indicating that EVs are more effective at infecting human primary fibroblast cells compared to naked viruses and are, therefore, crucial for MCPyV infection. Additionally, our TEM data analysis showed dense MCPyV virus-like particles largely accumulated inside of double membrane compartments in nHDF cells at day 4 post-infection (S4 Fig in S1 Appendix).

To confirm the presence of encapsidated virus inside of EVs, viral supernatants were extracted with chloroform [28] and/or sequentially treated with DNAse I [29] (Fig 4E). Chloroform extraction, which solubilizes lipids and EV membranes, had no effect on MCPyV genome detection in viral supernatants. However, DNAse I treatment greatly reduced MCPyV DNA, indicating the presence of capsid-free MCPyV genomes in the supernatant. When DNase I treatment was conducted following chloroform extraction, MCPyV DNA was only slightly decreased, but was still detectable, confirming that intact viral capsids, some of which were enclosed inside of EVs, protected MCPyV genomes from degradation. Moreover, MCPyV supernatants were treated with chloroform or Proteinase K to solubilize EV membranes or degrade viral capsids, respectively. Treated supernatants were then used to infect nHDF spheroids (Fig 4F). Chloroform treatment significantly reduced MCPyV infection, corroborating the importance of EVs for efficient MCPyV infection as seen in Fig 4D. Proteinase K treatment also partially prevented MCPyV infection, likely due to the degradation of naked virions, while keeping MCPyV-EVs intact for infection. This decrease in infection could also be attributed to the digestion of MCPyV virus particles that are tethered to the outside of EVs, and therefore not protected against Proteinase K treatment. Combination treatment of chloroform and Proteinase K completely ablated MCPyV infection, implicating the presence of intact infectious virions enclosed inside of EVs as MCPyV virus particles were no longer protected against Proteinase K digestion after the removal of EV membranes through chloroform extraction. To further investigate mechanisms of MCPyV viral entry, cells were pretreated with heparin, a known entry inhibitor of both MCPyV virions [6] and EVs [30] (Fig 4G). Heparin treatment significantly ablated MCPyV infection, emphasizing the requirement for heparin sulfate proteoglycans for the entry of both naked and EV-associated MCPyV virus particles.

### MCPyV utilizes EVs to evade antibody-mediated immune detection and modulate the innate immune response by altering cargo loading

**Fig 5:**
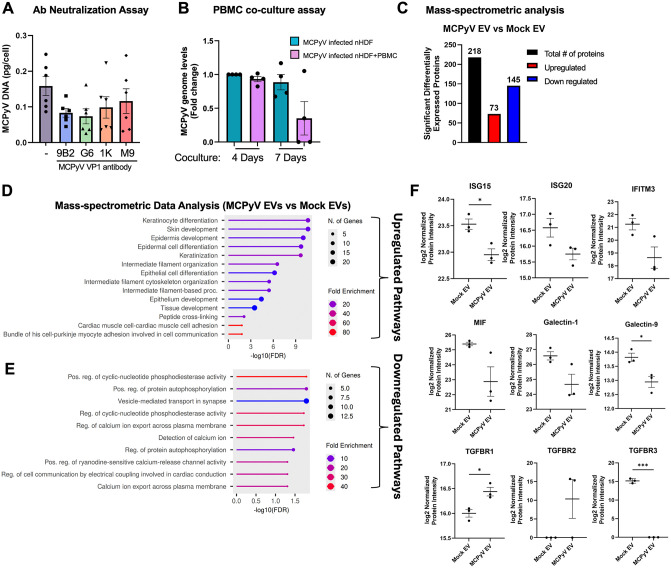
MCPyV hijacks extracellular vesicles to avoid immune surveillance. **(A)** Neutralization Assay. MCPyV-infected spheroids were incubated with anti-VP1 (9B2) or anti-VLP (G6, 1K, M9) antibodies to determine the efficacy of antibodies against VP1 capsids in neutralizing virus infectivity. MCPyV infection was partially inhibited by MCPyV capsid neutralizing antibodies (n = 6). **(B)** PBMC co-culture with MCPyV-infected nHDF cells in 3D. MCPyV-infected spheroids were generated using magnetic levitation and co-cultured with PBMCs for 4 or 7 days. nHDF spheroids were separated from PBMCs using magnetic force and then harvested. There was a partial reduction in MCPyV infection after 7 days of PBMC co-culture. PBMC co-culture with MCPyV-infected nHDF cells did not result in an immediate immune response, but required 7 days to reduce viral load, suggesting a delay in the immune response. **(C)** Mass spectrometry analysis of MCPyV-EVs. nHDF spheroids were transfected with an empty vector or the MCPyV genome. After 4 days, the 10,000 x g EV pellet was isolated from spheroid supernatants and subjected to mass spectrometry proteomic analysis (n = 3). The number of significant differentially expressed proteins packaged into mock- or MCPyV-EVs are reported. Red = upregulated, blue = downregulated proteins. **(D-E)** Gene Ontology pathway enrichment analysis on upregulated **(D)** or downregulated proteins **(E)** from MCPyV-EVs compared to Mock-EVs. **(F)** Decreased packaging of antiviral proteins in MCPyV-EVs. The packaging of various crucial antiviral restriction factors and immune response modulators is decreased in MCPyV-EVs compared to mock-EVs determined by mass spectrometry analysis (n = 3). ISG15: Interferon-stimulated gene 15. ISG20: Interferon-stimulated gene 20. IFITM3: Interferon-induced transmembrane protein 3. MIF: Macrophage migration inhibitory factor. TGFBR: Transforming growth factor beta receptor. Unpaired two-tailed Student’s *t* test was used to test significance, and error bars represent standard error of the mean (SEM).

Extracellular vesicles have been hijacked by viruses as a means to circumvent immune detection as previously described [31]. Specifically, EVs can act as a barrier to prevent the binding of neutralizing antibodies to the viral capsid. To determine if MCPyV-EVs can modulate host immune surveillance, we developed three new antibodies (G6, 1K, M9) against MCPyV VLPs [8,32]. The ability of these VLP antibodies to neutralize MCPyV infection was assessed and compared to the 9B2 antibody previously generated against VP1 peptides [8]. Treatment with VLP or VP1 anti-sera partially reduced MCPyV infection by ~50% compared to a mouse IgG control antibody (Fig 5A). To determine whether EVs could provide protection against neutralizing antibodies, EV-enriched media (EV(+)) and EV(-) were incubated with anti-9B2 VP1 and anti-G6 VLP antibodies, which displayed the highest neutralization efficacy. Inhibition of both EV(+) and EV(-) supernatant infections was observed with the 9B2 and G6 antibodies, suggesting selective protection against certain MCPyV capsid antibodies (S5 Fig in S1 Appendix). Given that co-culturing virus-infected fibroblasts with peripheral blood mononuclear cells (PBMCs) results in a robust immune response primarily driven by the recognition of infected cells by specific cytokines [33–36], we co-cultured PBMC with MCPyV-infected nHDF. While the initial PBMC response to a virus in co-culture takes place significantly earlier, often within 24–48 hours [35,37], PBMC co-culture did not result in an immediate response to MCPyV-infected nHDF cells. Instead, the immune response was delayed, requiring 7 days of co-culture before the viral load was reduced (Fig 5B).

It has been reported that viruses can modulate the contents of EVs to enhance or reduce the susceptibility of nearby cells to infection [31]. We then investigated how MCPyV could alter the EVs through mass spectrometry analysis. The 10K EV fraction, the fraction where we observed the MCPyV virion interaction with EVs, from mock- or MCPyV-infected cells was subjected to proteomic analysis, identifying ~6000 proteins (S1 Table). Specifically, statistical analysis identified 218 differentially packaged proteins with 145 proteins being significantly downregulated and 73 proteins being significantly upregulated in MCPyV-EVs compared to EVs isolated from uninfected nHDF cells (Mock EV) (Fig 5C). Pathway enrichment analysis using ShinyGO [38] identified that MCPyV-EVs were upregulated in pathways involved in skin development and epithelial cell differentiation, largely resulting from the increased expression of various keratin proteins compared to nHDF-EVs (Fig 5D and S1 Table). The regulation of keratinocyte differentiation is essential for HPV replication and viral gene expression [39], and thus implicates the importance of skin differentiation for MCPyV as well. Although most downregulated proteins fell into pathways mainly related to calcium transport or protein autophosphorylation (Fig 5E), we observed that MCPyV-EVs were packaged with fewer antiviral signaling proteins such as interferon-stimulated gene 15 (ISG15), ISG20, interferon-induced transmembrane protein 3 (IFITM3), and galectin proteins [40] compared to the antiviral gene profile in mock EVs (Fig 5F and S1 Table). Additionally, macrophage migration inhibitory factor (MIF), a cytokine that promotes inflammation and immune cell recruitment [41], was also decreased in MCPyV-EVs. We also identified a dysregulation of transforming growth factor beta receptors (TGFBR) packaged into EVs. TGFBR1 and TGFBR2 were found to be upregulated in MCPyV-EVs, whereas TGFBR3 was downregulated. TGF-β often signals through TGFBR1 and TGFBR2, developing an immunosuppressive microenvironment through the differentiation and expansion of regulatory T (Treg) cells and the inhibition of macrophage and dendritic cell function [42]. TGFBR3 can act as a negative regulator of TGF-β signaling by sequestering TGF-β from binding to TGFBR2 [43].

### Modulation of Wnt/MMP/Actin cytoskeletal signaling drives MCPyV infection in skin fibroblasts

**Fig 6:**
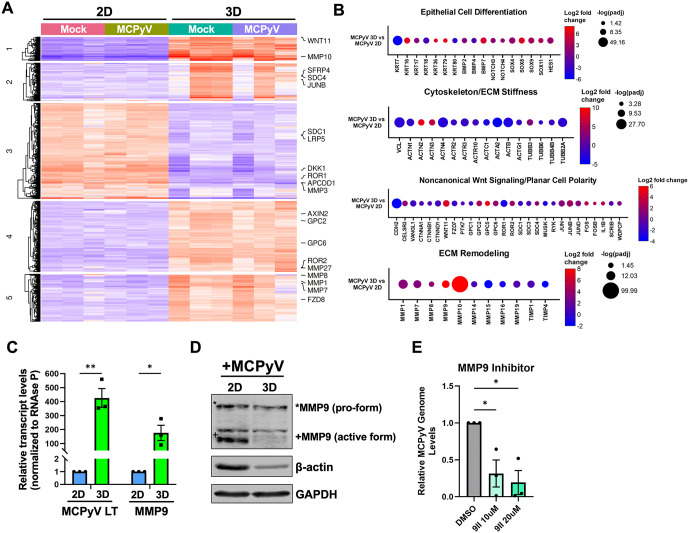
Activated Wnt signaling in the 3D microenvironment resembles skin wound healing processes in primary skin fibroblasts to promote enhanced MCPyV replication. **(A)** Gene expression changes of MCPyV-infected nHDF cells in 2D and 3D culture. RNA sequencing analysis was conducted on mock- or MCPyV-infected nHDF cells cultured as monolayers (2D) or spheroids (3D) at 4 days post-infection (n = 3). K means clustering (k = 5) heatmap comparing the top 2000 differentially expressed genes (DEG) of all 4 samples (Mock 2D, MCPyV 2D, Mock 3D, and MCPyV 3D). Only genes with a log2 fold change ≥ 1.5 and padj < 0.05 were retained for analysis using iDEP. Genes related to the canonical or noncanonical Wnt signaling pathway are annotated on the heatmap. **(B)** Activation of Wnt-driven pathways in 3D culture. Bubble plots of genes associated with pathways involving epithelial cell differentiation, cytoskeleton/extracellular matrix (ECM) stiffness, noncanonical Wnt signaling/planar cell polarity, or ECM remodeling in MCPyV-infected 3D spheroid culture compared to 2D monolayer culture. **(C)** Verification of Wnt signaling activation in 3D spheroids. RT-qPCR analysis of MCPyV-transfected nHDF cells cultured as a monolayer or spheroid 4 days post-transfection (n = 3). Both MCPyV LT and MMP9 RNA transcripts were increased verifying enhanced MCPyV gene expression and Wnt-driven MMP9 activation in the 3D microenvironment. RNAse P was used as a housekeeping gene. Unpaired two-tailed Student’s *t t*est was used to test significance, and error bars represent standard error of the mean (SEM). **(D)** Immunoblot analysis of MMP9 and β-actin. GAPDH was used as a loading control. The active form of MMP9 and β-actin cytoskeleton protein levels were decreased in the 3D microenvironment compared to 2D at 4 days post-MCPyV infection (n = 3). * = MMP9 pro-form. + = MMP9 active form. **(E)** MCPyV infection is regulated by MMP9. MCPyV-infected nHDF 3D spheroids were treated with DMSO or MMP9 inhibitor II (9II) for 4 days (n = 3). The 9II specifically targets the active functional form of MMP-9 [64] and inhibited MCPyV infection in a dose-dependent manner. Statistical significance was analyzed using a one-way analysis of variance (ANOVA) with Dunnett’s multiple-comparison tests.

Lastly, we wanted to interrogate the mechanism behind the enhanced MCPyV replication and infection we observed in 3D culture. We conducted RNA-Seq analysis on mock- or MCPyV-infected nHDF cells grown in 2D or 3D cultures. We found drastic changes in gene expression when comparing 3D to 2D culture (~11,000 differentially expressed genes (DEGs)), which was markedly higher than the number of DEGs (~600 DEGs) found between the same culturing method regardless of MCPyV infection status (Fig 6A and S2 Table). Through ShinyGO pathway enrichment, we discovered that pathways involved in the immune response such as “JAK-STAT signaling” and “MAPK signaling” were downregulated in MCPyV-infected spheroids compared to mock-infected spheroids (S6A Fig in S1 Appendix). These results reaffirm the data from Fig 5 that MCPyV can dysregulate the immune response. Pathways upregulated by 3D MCPyV-infection included “p53 signaling”, recapitulating previous results [20,44], and “neurogenerative diseases”. Using Gene Ontology (GO) pathway analysis, we found that many of the enriched pathways in MCPyV-infected spheroids compared to MCPyV-infected monolayers were related to the “epithelial cell differentiation”, “cytoskeleton/extracellular matrix (ECM) stiffness”, “noncanonical Wnt signaling/planar cell polarity”, and “ECM remodeling” (Figs 6B and S6B in S1 Appendix). The majority of cytoskeletal genes, namely β-actin and actin-related genes, were found to be downregulated in 3D spheroids compared to 2D monolayers. Decreased β-actin transcript and protein levels in infected 3D spheroids compared to 2D monolayers was confirmed via RT-qPCR and western blot analysis (S7A Fig in S1 Appendix, Fig 6D).

Importantly, further analysis using iDEP [45] highlighted numerous DEGs involved in canonical or noncanonical Wnt signaling (Fig 6A). Specifically, we observed an upregulation of Wnt11 and an enrichment in the “transcription factor AP-1 complex” pathway (S3 Table), which includes an increased expression of Jun family transcription factors (Fig 6B), implying an activation of noncanonical planar cell polarity Wnt signaling [46]. Examining downstream of Wnt signaling [47], we observed increases in MMP1, MMP7, MMP8, MMP9, MMP10, and MMP19 transcripts (Fig 6B). Elevated levels of MMP9 transcripts were found in cells transfected with the MCPyV genome when grown in 3D compared to 2D by RT-qPCR (Fig 6C). Interestingly, we observed a decrease in the intracellular active form of MMP9 in 3D compared to 2D, suggesting MMP9 secretion (Fig 6D). Indeed, we observed an increase in extracellular MMP9 expression from 3D conditioned media compared to 2D conditioned media (S7B Fig in S1 Appendix). MMP9 secretion, subsequent matrix degradation, and actin rearrangement in the 3D microenvironment, signify ongoing active tissue remodeling processes. Importantly, we observed a decrease in MCPyV infection after treatment with the MMP9 inhibitor 9II (Fig 6E) [48], a selective inhibitor that targets the active form of MMP9. To determine how MMP9 regulates MCPyV replication, we pretreated spheroids with 9II for 1 hour and then infected the spheroids for 1 hour, or treated MCPyV-infected spheroids 1 day post-infection with 9II for 4 days to ascertain if MMP9 is involved in viral entry or post-entry replication, respectively (S7C Fig in S1 Appendix). MMP9 inhibition did not affect viral entry or post-entry viral replication. Only when cells were infected and treated with 9II at the same time, before spheroids formed, did MMP9 inhibition reduce MCPyV infection. This suggests that the breakdown of the ECM inside of the spheroid is required for efficient MCPyV virion transmission. These data reaffirm that Wnt/MMP signaling may play a critical role in regulating the MCPyV lifecycle and viral infection.

## Discussion

Uncovering aspects of the MCPyV lifecycle and host pathogen interactions have been challenging due to a lack of advanced *in vitro* models and strict host range. Though the current use of quasiviruses produced from HEK293 cells have provided significant contributions to the field regarding virus entry [6,49], immune response [50,51], and tropism [5,52], these recombinant viruses do not fully recapitulate the skin infection or host range of naturally produced MCPyV virions. In this study, we developed an innovative 3D system that can support enhanced MCPyV replication, and subsequently, produce native MCPyV virions from primary human skin fibroblasts, which have been identified as the natural host cells of MCPyV. Of note, our virus supernatants were generated by using the MCPyV genome alone without exogenous complementation of MCPyV viral proteins or modulation of culture media components to accurately mimic natural infection conditions. We observed that native virions produced from 3D spheroids have a restricted host range with higher infection efficiency in nHDF cells compared to HEK293 cells (Fig 3H). Since previous studies have used recombinant MCPyV virions to infect HEK293 cells to investigate the viral lifecycle, our data highlights potential distinctions in host tropism between native and recombinant MCPyV virions. Further investigation of the host range and viral lifecycle for native MCPyV virions derived from natural host cells will be required to clarify differences between native and recombinant virions.

Interactions between cells and the extracellular matrix or the physical structure of skin dermal fibroblasts might create a microenvironment that is conducive to MCPyV replication since planar cell polarity, cytoskeletal rearrangement, and cell morphology pathways were modulated as observed in our RNA seq analysis. This skin environment is often driven by active core Wnt signaling, creating asymmetric actin dynamics that physically reprograms tissue during wound repair [53]. Our study showed that increased active MMP9 expression and disrupted actin dynamics, in combination with changes in cell polarity or shape allowed enhanced virion release and efficient viral spread. Similarly, a previous study has shown that changes in culture conditions by addition of GSK-3β inhibitor for activated Wnt signaling and collagenase IV to mimic MMP expression were necessary to grant enhanced access of MCPyV virions to the dermal fibroblasts for infection [5]. In our study, upregulation of Wnt pathway genes in 3D culture did not require any cell culture media modulation, highlighting the importance of the inherent physiology of the skin microenvironment and natural environment for Wnt signaling activation to support natural virus egress or localized cell-to-cell transmission, enhancing overall viral pathogenesis.

Skin wound repair is a complex process that while generally effective throughout life, it significantly deteriorates with age [54]. Our RNA-seq data analysis revealed alterations in epithelial cell differentiation genes in the 3D microenvironment compared to 2D monolayer cell culture conditions, indicating that our 3D model may recapitulate skin wound healing processes. Wnt signaling activation during cutaneous wound repair is a critical driver of epithelial cell differentiation, regeneration, and neural differentiation [55,56], often regulating the transition between stemness and mature cell types. During this process, various kinds of cells are reprogrammed to proliferate and differentiate, thereby restoring the skin’s barrier and regenerating the skin. Defects in the skin wound repair process due to aging or chronic exposure to UV radiation might promote enhanced access of the dermal layer of the skin for efficient MCPyV entry and infection, contributing to MCPyV pathogenesis.

Viruses have been reported to utilize EVs to modulate the cell’s susceptibility to infection, enhance virulence, and evade the immune response [31,57]. Our study revealed that MCPyV utilizes EVs for normal skin cell infection with restricted host range (Fig 3H). MCPyV virions isolated from HEK293 in previous studies were only detected as non-enveloped naked structures by TEM analyses [8,58,59]. We observed that one or more MCPyV virions were tethered or packaged into EVs (Fig 4A), which can increase the multiplicity of infection, augmenting infectivity by evading host immunity. Similarly, previous reports have shown that other pathogenic human polyomaviruses, such BK polyomavirus (BKPyV) and JC polyomavirus (JCPyV) utilize EVs for infection [60,61]. JCPyV- and BKPyV-associated EVs were observed to contain five or more virions per EV when derived from natural host cell lines such as glial or renal cells, respectively [60,61]. Extracellular vesicle (EV)-associated viruses can delay the host immune response by shielding virions within host-derived lipid membranes, allowing them to evade neutralizing antibodies and immune recognition. Partial inhibition of infection was observed with treatment of MCPyV capsid anti-sera (Figs 5A and S5 in S1 Appendix), suggesting that virions enclosed inside of EV membranes were protected against neutralizing antibodies. Virus particles tethered to the outside of EV membranes could still be subjected to neutralization and thus confer sensitivity to MCPyV capsid anti-sera as observed in the EV(+) infection conditions. The delay of host immune response by MCPyV-EVs also aids the virus to remain “hidden” while building a reservoir (Fig 5B), allowing it to escape the initial, weak immune response and establishing a long-term, persistent infection. Moreover, MCPyV decreased the packaging of various antiviral proteins, such as ISGs, into EVs (Fig 5F and S1 Table). These data suggest MCPyV-infected cells could enhance the susceptibility of infection by inducing an immunosuppressive environment that allows pathogens to avoid immune detection and facilitate the spread of virions to neighboring cells. The abundance of β-catenin in MCPyV-EVs’ cargo loading was found to have increased by 5% compared to the mock-EVs (S1 Table), which implies a modest, yet potentially significant shift in the balance of Wnt signaling activation during MCPyV-EV infection. Further research is needed to examine the importance of EVs as a critical mechanism for immune evasion and pathogenesis of human cancer viruses.

**Fig 7:**
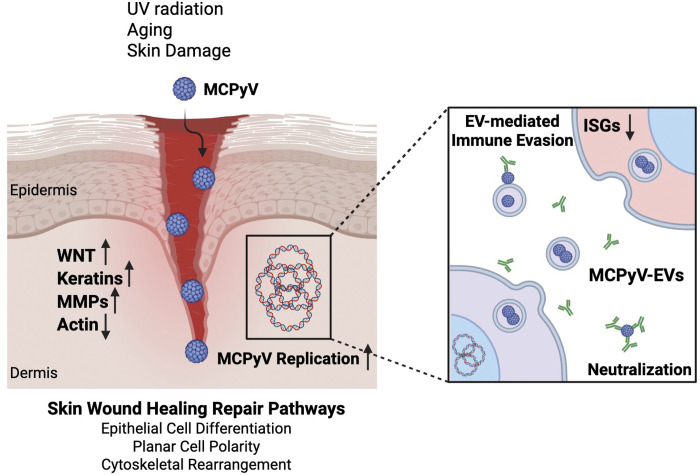
MCPyV skin infection and EV-mediated host immune evasion during skin damage and repair processes. MCPyV-infected primary human dermal fibroblast cells support enhanced viral gene transcript expression, genome replication, and persistence when cultured in a 3D spheroid microenvironment that resembles damaged skin with active Wnt signaling and matrix metalloprotease (MMP) expression. Changes in epithelial cell differentiation, planar cell polarity, or cytoskeletal rearrangement could remodel the skin architecture including dermal fibroblasts and may regulate the MCPyV lifecycle. Enhanced MCPyV replication enables the release of both naked MCPyV virions or virus particles that are associated with extracellular vesicles (MCPyV-EVs). EVs protect MCPyV virions against neutralizing antibodies and carry reduced levels of active antiviral factors (such as ISGs) that suppress immune responses and promote efficient viral transmission. Figure created in BioRender (https://BioRender.com/ttjekf3).

In conclusion, the possibility of EVs as a mode of infection reveals a fundamental mechanism of the MCPyV lifecycle and immune evasion, which was previously unknown and could potentially explain difficulties in the virus’s detection both *in vitro* and *in vivo*. These findings underscore the significance of investigating the extent to which EVs and the active Wnt-driven microenvironment during skin damage and aging are involved in MCPyV infection (Fig 7) [62,63].

## Materials and methods

Detailed experimental procedures and information on materials used are provided in the SI Appendix. Statistical analysis was conducted using GraphPad Prism 10.4.2 Software. Figures represent average value with error bars displaying standard error. Unpaired two-tailed Student’s *t* test, unpaired multiple Student’s *t* test using the Holm-Sidak method, one-way analysis of variance (ANOVA) with Dunnett’s multiple-comparison tests, and two-way ANOVA analyses with Fisher’s LSD, Dunnett’s, or Tukey’s multiple-comparison tests were utilized to determine statistical significance, with p < 0.05 considered significant.

## Supporting information

S1 Appendix Detailed description of experimental methodology and supplementary figures. (DOCX)

S1 Table Mass spectrometric analysis of MCPyV-EVs. (XLSX)

S2 Table RNA-seq analysis of MCPyV-EV infected nHDF cells. (XLSX)

S3 Table Integrated differential expression and pathway analysis of RNA-seq data. (XLSX)

S4 Table Antibodies used in this study. (XLSX)

S5 Table Primers used in this study. (XLSX)
