# Supplementary material for: Merkel cell polyomavirus exploits extracellular vesicles for skin infection and host immune evasion through activated Wnt signaling
Source: PLoS Pathog. 2026 Jun 23;22(6):e1014360. doi: 10.1371/journal.ppat.1014360 (PMC13289936; doi:10.1371/journal.ppat.1014360)
Supplement: S1 Appendix — (DOCX) [file ppat.1014360.s001.docx]

Supplementary Information for

**Merkel Cell Polyomavirus Exploits Extracellular Vesicles for Skin Infection and Host Immune Evasion Through Activated Wnt Signaling**

**Short Title: Activated Wnt Signaling facilitates EV-associated Merkel Cell Polyomavirus Skin Infection**

Alexander M. Pham^1,2^, Luz E. Ortiz^1,2^, Han Chen^3^, Neil Christensen^4^, Hyun Jin Kwun^1,2,5*^

^1^Department of Cell and Biological Systems, Penn State University College of Medicine, Hershey,

[Pennsylvania](https://www.bing.com/ck/a?!&&p=33c99c5f936089be18b1b66859996ee24fd06c12d5a971a08394ce1281cdae85JmltdHM9MTc4MDk2MzIwMA&ptn=3&ver=2&hsh=4&fclid=1867bdf5-8ba2-68e7-0aab-aada8ae669f0&psq=PA+17033%2c+USA&u=a1aHR0cHM6Ly93d3cuemlwZGF0YW1hcHMuY29tLzE3MDMz&ntb=1), United States of America

^2^Penn State Cancer Institute, Hershey, [Pennsylvania](https://www.bing.com/ck/a?!&&p=33c99c5f936089be18b1b66859996ee24fd06c12d5a971a08394ce1281cdae85JmltdHM9MTc4MDk2MzIwMA&ptn=3&ver=2&hsh=4&fclid=1867bdf5-8ba2-68e7-0aab-aada8ae669f0&psq=PA+17033%2c+USA&u=a1aHR0cHM6Ly93d3cuemlwZGF0YW1hcHMuY29tLzE3MDMz&ntb=1), United States of America

^3^Transmission Electron Microscopy Core, Penn State College of Medicine, Hershey, [Pennsylvania](https://www.bing.com/ck/a?!&&p=33c99c5f936089be18b1b66859996ee24fd06c12d5a971a08394ce1281cdae85JmltdHM9MTc4MDk2MzIwMA&ptn=3&ver=2&hsh=4&fclid=1867bdf5-8ba2-68e7-0aab-aada8ae669f0&psq=PA+17033%2c+USA&u=a1aHR0cHM6Ly93d3cuemlwZGF0YW1hcHMuY29tLzE3MDMz&ntb=1), United States of America

^4^Department of Pathology and Laboratory Medicine, Hershey, [Pennsylvania](https://www.bing.com/ck/a?!&&p=33c99c5f936089be18b1b66859996ee24fd06c12d5a971a08394ce1281cdae85JmltdHM9MTc4MDk2MzIwMA&ptn=3&ver=2&hsh=4&fclid=1867bdf5-8ba2-68e7-0aab-aada8ae669f0&psq=PA+17033%2c+USA&u=a1aHR0cHM6Ly93d3cuemlwZGF0YW1hcHMuY29tLzE3MDMz&ntb=1), United States of America

^5^Penn State Clinical and Translational Science Institute, Hershey, [Pennsylvania](https://www.bing.com/ck/a?!&&p=33c99c5f936089be18b1b66859996ee24fd06c12d5a971a08394ce1281cdae85JmltdHM9MTc4MDk2MzIwMA&ptn=3&ver=2&hsh=4&fclid=1867bdf5-8ba2-68e7-0aab-aada8ae669f0&psq=PA+17033%2c+USA&u=a1aHR0cHM6Ly93d3cuemlwZGF0YW1hcHMuY29tLzE3MDMz&ntb=1), United States of America

*****Corresponding author: Hyun Jin Kwun, Ph.D.

Email: hxk479@psu.edu

**This PDF file includes:**

Materials and Methods

S1 Appendix S1-S7 Figures

Supporting References

S1 Appendix S1-S5 Tables

**Materials and Methods**

**Cell Culture, Plasmids and Antibodies**

Neonatal primary human dermal fibroblasts (nHDF, Lonza), adult primary human dermal fibroblast (aHDF, Lonza), HEK293 (Sigma-Aldrich), and BJ-hTERT cells [1] were cultured in Dulbecco’s modified Eagle’s medium (DMEM) with 10% premium grade fetal bovine serum (FBS, Seradigm). Primary cells used in this study were grown up to passage 25. Wild type and E3 ligase mutant MCPyV [2] were cloned into minicircle vectors and generated as previously described [1, 3]. Antibodies against virus-like particles (VLP) were generated (1K, M9, G6) at the Custom Antibody Core (Penn State College of Medicine) and MCPyV VP1 antibody 9B2 was kindly provided by Dr. Chang and Dr. Moore (University of Pittsburgh). All antibodies used in this study are listed in Supplementary **S4 Table**.

**MCPyV Virion Production from primary dermal fibroblasts**

For MCPyV virion production, 1×10^6^ nHDF cells (<passage 25) were reverse-transfected with 10 µg of empty vector or MCPyV minicircle genome [1] using jetOPTIMUS reagent (Polyplus) and seeded into low attachment plates (Greiner Bio-One). Cell culture medium (DMEM with 10% FBS) was changed with fresh medium at 24 hours after transfection. At 4 days post-transfection, the supernatant of transfected cells was collected and filtered with a 0.45 µm syringe filter for virus infection.

**MCPyV Infection and MCPyV Genome Analysis**

A standard curve was generated by running qPCR on serial dilutions of known quantities of MCPyV genomes for virus quantification. Then, ~100 µL of the filtered mock (empty vector) or viral supernatant (2 x 10^8^ or 2 x 10^9^ MCPyV genome copies) was used to infect nHDF cells cultured as a monolayer (5 x 10^4^ cells seeded into a 24-well adherent plate) or spheroids (5 x 10^4^ cells seeded into a 96-well low attachment plate) at the time of seeding. Cell culture media was supplemented with or without polybrene (Sigma-Aldrich, 12 µg/mL) and infected cells were harvested 4 days post-infection. Cells were also harvested at 2 days or 7/14 days after seeding for early replication and genome maintenance experiments, respectively. MCPyV viral DNA was isolated using a Quick-DNA Miniprep Kit (Zymo Research). To detect viral genome levels, 20 ng of DpnI-digested DNA was used for qPCR using PowerUp SYBR Green Master Mix (Applied Biosystems) containing uracil-DNA glycosylase (UDG) and dUTP, according to the manufacturer’s instructions. Quantitative analyses were performed using the comparative ΔΔCt method by detecting ribonuclease P (RNase P) as a reference gene and MCPyV detection primer pairs listed in **S5 Table**. All qPCR experiments included melting curve analyses to confirm the specificity of the amplicons (95°C for 15 s, 60°C for 20 s, and 95°C for 15 s).

**3D spheroid formation using primary fibroblasts**

Human fibroblast cells (nHDF, aHDF, and BJ-hTERT) were reverse-transfected with 10 µg of MCPyV minicircle DNA using jetOPTIMUS (Polyplus) following the manufacturer’s protocol and seeded into 6-well plates. For magnetic 3D bioprinting, one day after seeding, 30 µL of NanoShuttle-PL (Greiner Bio-One) was added to each well and incubated overnight. Then, magnetized cells (5 x 10^4^ cells/well) were seeded into a 24-well adherent plate or a 24-well low attachment surface plate (Greiner Bio-One) to prevent cell attachment. To generate spheroids using magnetic levitation, the low attachment plate was placed upon a 24-well concentration magnetic drive plate (Greiner Bio-One) for one day. The magnet below the wells aggregates the magnetized cells into a defined 3D shape. Spheroids were maintained in low attachment surface plates without the magnetic drive plate until harvest. For hanging drop spheroids, 5 x 10^4^ cells (in ~30 µL of media) were pipetted onto the inside of the lid of a 10 cm^2^ plate and 8 mL of PBS was added to the bottom of the plate. The lid containing the cell droplets was inverted and placed onto the top of the plate. The hanging drop spheroids were maintained inverted on the lid until harvest. To generate spheroids using suspension culture, 5 x 10^4^ cells were seeded in 96-well U bottom low attachment plates (Greiner Bio-One).

**RT-qPCR**

Total RNA was extracted from monolayer or spheroid cells 4 days post-transfection using TRI Reagent (Sigma-Aldrich) following the manufacturer’s protocol. RNA was treated with DNAse I (NEB) for 15 minutes at 37°C and then incubated at 65°C for 10 minutes to heat inactive the DNAse I. To measure viral, MMP9, or β-actin transcripts, 25 ng of RNA was used for RT-qPCR and detected using an iTaq Universal One-Step RT-qPCR Kit (Bio-Rad). Quantitative analyses were performed using the comparative ΔΔCt method by detecting RNase P as a reference gene and using primer pairs listed in **S5 Table**. All RT-qPCR experiments included melting curve analyses to confirm the specificity of the amplicons (95°C for 15 s, 60°C for 20 s, and 95°C for 15 s).

**Immunofluorescence Assay**

Primary nHDF cells were seeded into 8-well chamber slides and then infected with supernatants with polybrene (12 µg/mL). After 4 days, cells were fixed with 2% paraformaldehyde in PBS (Alfa Aesar) for 15 minutes and permeabilized with 0.5% Triton X-100 (Sigma-Aldrich) for 20 minutes. Cells were then blocked with 5% bovine serum albumin (BSA) and stained with the following primary antibodies: mouse MCPyV VP1 (CM9B2, 1:500) and MCPyV LT (CM2B4, Santa Cruz, 1:2000) or Alexa Fluor 647-conjugated MCPyV LT (CM2B4, Santa Cruz, 1:2000) for 1 hour at room temperature (RT). Primary antibodies were detected by incubating Alexa Fluor 488-conjugated goat anti-mouse IgG (H+L Highly Cross Adsorbed, Invitrogen, 1:4000) secondary antibodies for 30 minutes at RT. Cells were counterstained with DAPI (Thermo Scientific, 0.5 µg/mL) for 10 minutes. Coverslips were mounted using Vectashield Plus Antifade Mounting Medium (Vector Laboratories) and imaged using a REVOLVE4 microscope (Echo Laboratories).

**Fluorescence in Situ Hybridization (FISH) Detection of MCPyV genome**

FISH was performed using a Cy5-labeled DNA probe complementary to the MCPyV core origin sequence (**S5 Table**). Cells were fixed with 4% paraformaldehyde in PBS for 10 mins at RT, followed by 3x wash steps of 0.3 M glycine (Sigma-Aldrich) in PBS for 5 minutes. Cells were permeabilized in 1X permeabilization buffer (Invitrogen) for 10 min at RT, digested with 5 µg/ml Proteinase K (Fisher Scientific) in 0.1% Tween-20 in PBS for 10 mins at 37°C, and followed by a washing step with 2X saline-sodium citrate (SSC) pH 7.0 (Invitrogen). Hybridization mixture containing 0.5 µM Cy5-labeled MCPyV probe, 2 ng sonicated salmon sperm (Agilent Technologies) in 60% deionized formamide, 10% dextran sulfate sodium salt, and 2X SSC (pH 7.0) was added to the cells and denatured for 5 mins at 90°C. Immediately afterwards, slides were placed in a humidity chamber at 37°C overnight. After hybridization, cells were stringently washed in 2XSSC pH 7.0 at 70°C for 2 minutes, followed by washes with 2XSSC and PBS. Slides were protected from the light from this point forward. To continue with the immunofluorescence staining, cells were blocked with 5% BSA for 1 hour at RT and incubated with primary antibody CM2B4 (Santa Cruz Biotechnology, 1:500) overnight at 4°C. Cells were then incubated with Alexa Fluor 488-conjugated goat anti-mouse IgG secondary antibody for 1 hour at RT and counterstained with 0.5 µg/ml DAPI for 10 mins.

**OptiPrep Density Centrifugation**

Primary nHDF cells transfected with 10 µg of wild type MCPyV minicircle were cultured as a monolayer or as spheroids. Four days post-transfection, cells were harvested and resuspended in one cell pellet volume of PBS containing 9.5 mM MgCl_2_, 25 mM ammonium sulfate, 0.5% Triton X-100, 0.1% benzonase (Sigma-Aldrich), 1 mM ATP, 0.1% ATP-dependent DNAse (Biosearch Technologies), and 1x antibiotic-antimycotic (Corning) and left overnight at 37°C. The lysate was then placed on ice and 0.17 volume of 5 M NaCl was added. The lysate was clarified by spinning at 12,000 x g for 10 minutes at 4°C. The clarified lysate was added to a fresh tube, and the pellet was extracted once more using 2 volumes of PBS supplemented with 0.8 M salt. Clarified lysates were combined and overlaid on top of a 2.1 mL discontinuous OptiPrep (iodixanol, Sigma-Aldrich) gradient (0.7 mL of 27%, 0.7 mL of 33%, and 0.7 mL 39%). OptiPrep was diluted using the 0.8 M salt PBS solution. MCPyV virions were purified by centrifugation at 234,000 x g for 3.5 hours at 16°C in a TH-660 swing bucket rotor. Fractions (200 µL) were taken from the top and subjected to qPCR to assess MCPyV DNA levels.

**Differential Centrifugation for Extracellular Vesicle Isolation**

Extracellular vesicles (EV) were isolated and purified using differential centrifugation [4, 5]. MCPyV-transfected spheroids and associated culture media were spun at 500 x g for 10 minutes using a tabletop 5430R centrifuge (Eppendorf) to pellet cells. The supernatant was then centrifuged at 2,000 x g for 15 minutes to remove remaining cells or debris. The remaining supernatant was transferred to a polyallomer thin wall tube (Seton Scientific) and spun at 10,000 x g for 30 minutes or 100,000 x g for 2 hours to isolate large and small extracellular vesicles respectively using a Sorvall WX80+ ultracentrifuge (Thermo Scientific) with either a TH-660 or TH-641 swinging bucket rotor. Spheroid and EV pellets were washed and repelleted in sterile PBS by repeating the previous centrifugation conditions and stored at -20°C or -80°C for short-term or long-term storage respectively. Extracellular vesicle-depleted media (EV(-)) (**Fig 4D**) was obtained by isolating the supernatant after differential centrifugation without disturbing the 100,000 x g pellet.

**Immunoblot Analysis**

Spheroid pellets (500 x g and 2000 x g) were lysed with Pierce radioimmunoprecipitation assay buffer (RIPA, Thermo Scientific) supplemented with 1 mM PMSF and 1 mM benzamidine protease inhibitors for at least 1 hour at 4°C. Soluble fractions were isolated by centrifugation at 16,500 x g for 15 minutes at 4°C and then boiled at 95°C for 5 minutes. Extracellular vesicle suspensions (10,000 x g and 100,000 x g) were lysed using an equal volume of urea lysis buffer (5% SDS, 10 mM EDTA, 120 mM Tris-HCl pH 6.8, 9 M urea, 2.5% β-mercaptoethanol) [6] and shaken at RT for 15 minutes. Equal volumes of lysates were loaded for immunoblot analysis. Membranes were blocked with 5% milk in TBS with 0.1% Tween-20 and incubated with primary antibodies overnight at 4°C. Secondary antibodies were incubated for 2 hours at RT. Detection of protein was determined using a quantitative infrared (IR) imaging system, Odyssey CLX (LI-COR).

**Immunoprecipitation**

MCPyV-infected nHDF in 3D or 293 cells transfected with LT were lysed using a 1:1 ratio of IP buffer (50 mM Tris-HCl (pH 8.0), 150 mM NaCl, 1% TritonX-100):RIPA lysis buffer supplemented with 1 mM PMSF and 1 mM benzamidine protease inhibitors. Lysates and protein G Dynabead (Thermo Fisher) were incubated overnight at 4°C with mouse normal IgG (Cell Signaling) or CM2B4 antibody (Santa Cruz Biotechnology). The Dynabeads were washed and resuspended in 2X SDS loading buffer. LT was detected by SDS-PAGE using a 4–20% gradient precast Criterion TGX gel (BioRad) followed by immunoblot analysis with CM2B4 antibody.

**Chloroform and DNase I Genome Protection Assay**

Chloroform [7, 8] and DNase I genome protection assays were performed as previously described [9]. Briefly, chloroform (Fisher Scientific, 10%) and viral supernatants were incubated at RT for 10 minutes and then centrifuged at 1000 RPM for 10 minutes. The aqueous phase was isolated and filtered at 0.45 µm. DNase I (NEB, 120 Units/mL) was added to the MCPyV supernatant and placed in a shaking incubator at 37°C for 1 hour. DNase I was inactivated through incubation at 65°C for 10 minutes. For combined treatment of chloroform and DNase I, MCPyV EVs were first extracted with chloroform and then treated with DNase I. The presence of MCPyV DNA was then detected by qPCR.

**Chloroform and Proteinase K Treatment**

Viral supernatants were treated with PBS or chloroform (1:1 by volume) to extract extracellular vesicle membranes. Treated supernatants were incubated at RT for 10 minutes and then centrifuged at 1000 RPM for 10 minutes to isolate the aqueous layer. Supernatants were also treated with Proteinase K (NEB, 50 µg/mL) for 15 minutes on ice. 1 mM benzamidine was then added and incubated for 10 minutes on ice. For combined treatments, viral supernatants were first extracted with chloroform and then treated with Proteinase K. Supernatants were then filtered at 0.45 µm. To remove the chloroform and Proteinase K from the supernatant, viruses were precipitated using Viro-PEG (Oz Biosciences) according to the manufacturer’s instructions. MCPyV virus pellets were resuspended in 10% FBS in DMEM. Treated viral supernatants were used to infect nHDF spheroids for 4 days.

**Antibody Neutralization and Heparin Assays**

For the neutralization assays, 5 µg of mouse IgG or 500 µg total protein from 9B2, 1K, M9, or G6 hybridoma supernatant was pre-incubated with nHDF in 3D spheroids for 1 hour using a low attachment surface plate. Then, nHDF spheroids were infected with MCPyV (~2 x 10^8^ MCPyV genome copies/5 x 10^4^ nHDFs) for ~1 day. For heparin assay, spheroids were preincubated with DMSO or heparin (Sigma-Aldrich, 10 µg/mL, 50 µg/mL, 100 µg/mL) for 1 hour, and then cells were infected with MCPyV stocks (~2 x 10^8^ MCPyV genome copies/5 x 10^4^ nHDFs) for 1 hour.

**PBMC co-culture**

PBMCs isolated from 27 years old female normal patient (Discovery Life Sciences) were maintained in RPMI 1640 with 10% FBS for 4 days. nHDF cells were magnetized and infected with MCPyV for 24 hours in 3D using a low attachment surface plate. nHDF cells were formed into spheroids using magnetic levitation and then were co-cultured with PBMCs (2 x 10^5^). On day 4 and day 7 after coculturing, magnetized nHDF cell spheroids were separated from PBMC using magnetic force. nHDF spheroids were collected after three washes with PBS, then MCPyV viral DNA was isolated from the nHDF cell spheroids using a Quick-DNA Miniprep Kit (Zymo Research) and analyzed by qPCR analysis.

**Extracellular MMP9 Detection**

Primary nHDF cells were transfected with the MCPyV genome and cultured as a monolayer or spheroid. After three days, the media was replaced with serum-free media for overnight culture. The next day, the conditioned media was harvested and spun at 2,000 x g for 15 minutes and filtered with a 0.45 µm syringe filter to remove cellular debris. The conditioned media was supplemented with 1 mM PMSF and 1 mM benzamidine and concentrated using a 30 kDa MWCO centrifugal filter (Millipore). Loading of conditioned media for immunoblot analysis was normalized to cell count.

**MMP9 Inhibitor Treatment**

Spheroids were treated with DMSO or MMP9 inhibitor (9II, Sigma-Aldrich) [10] using three different regiments: **1)** spheroids were pre-incubated with 9II for 1 hour and then infected with MCPyV viral supernatants to examine viral entry, **2)** spheroids were infected with viral supernatants for 1 day and then treated with 9II for 4 days to examine post-entry replication, and **3)** spheroids were infected and treated with 9II for four days to examine the impact of ECM on viral transmission in the spheroid microenvironment. After two days, half the cell culture media was replenished with fresh media.

**Transmission Electron Microscopy**

Cells infected with MCPyV for 4 days in suspension spheroid culture were harvested and fixed with 2.5% glutaraldehyde and 2% paraformaldehyde in 0.1 M phosphate buffer (pH 7.4) and further fixed in 1% osmium tetroxide in 0.1 M phosphate buffer (pH 7.4) for 1 hour. Samples were dehydrated in a graduated ethanol series: pure acetone embedded in LX-112 (Ladd Research). Thin sections (70 nm) of spheroids (one third of the distance from the top) were stained with uranyl acetate and lead citrate and imaged at 60 kV using a JEOL JEM1400 Transmission Electron Microscope (JEOL USA Inc.) For negative staining, the 10,000 x g EV pellet was fixed with 2% paraformaldehyde overnight at 4°C. Samples were adsorbed to a Formvar carbon-coated 400-mesh nickel grid (Electron Microscopy Sciences) for one minute and negatively stained with 1% uranyl acetate.

**Mass Spectrometry Analysis of Extracellular Vesicles**

Primary nHDF cells were transfected with the MCPyV genome and cultured in low attachment surface plates (Greiner Bio-One). Cell media was changed with DMEM and 10% exosome-depleted FBS (Gibco) at one day post-transfection. Supernatant was collected at 4 days post-transfection. The 10,000 x g extracellular vesicle pellets were isolated from mock- or MCPyV-infected spheroids for mass spectrometry analysis (MS Bioworks). Samples were lysed with RIPA buffer (2% SDS, 10 mM NaCl, 50 mM Tris HCl pH 8), and protein concentration were quantified using a Qubit protein assay (Invitrogen). 10 µg of protein was processed by sodium dodecyl sulfate‐polyacrylamide gel electrophoresis (SDS-PAGE) using a 10% Bis-Tris NuPage mini gel for in-gel digestion. Proteins were washed with 25 mM ammonium bicarbonate followed by an acetonitrile wash. Samples were reduced with 10 mM dithiothreitol at 60°C and alkylated with 50 mM iodoacetamide at RT. Trypsin digestion was conducted at 37°C for 4 hours and quenched with formic acid. Samples were then suspended in 0.1% trifluoroacetic acid. Then, samples were analyzed by a nano LC/MS with Vanquish Neo nanoscale UPLC system interfaced to an Orbital Astral (ThermoFisher). Peptides were loaded onto an IonOpticks 25 cm x 75 µm Aurora Ultimate analytical column at 350 nL/min at 55°C. The mass spectrometer was operated in data-independent mode. Sequentially, full scan MS data (240,000 FWHM resolution) from m/z 380-980 was followed by 300 x 2m/z precursor isolation windows and products were acquired on the Astra at 40,000 FWHM resolution. The maximum injection time was set to 3.5 ms for DIA and the NCE was set to 25. The data was analyzed using DIA-NN 2.2.0 followed by analysis in Perseus for quantification of protein abundance, log2 normalization, and statistical analysis. Only proteins with one or more peptides and a false discovery rate (FDR) below 0.01 were retained for analysis. ShinyGO [11] pathway enrichment analysis was used on proteins significantly upregulated or downregulated in MCPyV-EVs compared to mock-infected EVs and reported pathways had a FDR value < 0.05.

**RNA-Seq Analysis of MCPyV-infected Primary Skin Dermal Fibroblasts**

RNA was extracted using TRI Reagent (Sigma-Aldrich) from mock- or MCPyV-infected monolayers or spheroids at 4 days post-infection. Monolayer infections occurred in the presence of polybrene. Transcript reads were aligned to the human genome (hg38) (Novogene). Differential gene expression was obtained using DESeq2 1.42.0 with the following parameters (log2(fold change) ≥ 1, padj ≤ 0.05). Identified pathways using Gene Ontology (GO) or Kyoto’s Encyclopedia of Genes and Genomes (KEGG) pathway enrichment analysis had FDR values < 0.05. The RNA seq dataset is publicly available at the NCBI Gene Expression Omnibus (GEO) with the following accession number: GSE311065. Further analysis using integrated differential expression and pathway (iDEP) [12] using raw gene counts was also conducted. Data was preprocessed to only include genes with a count per million (CPM) ≥ 0.25 in at least one sample and transformed using rlog. Differential gene expression was conducted using DESeq2 (Fold change > 1.5, padj < 0.05) and the top 2000 DEGs were utilized to generate a heatmap using k means clustering (k = 5). Pathway analysis was filtered by FDR < 0.05.

**S1 Fig. Normal skin fibroblasts support efficient MCPyV early and late gene transcription.** **(A)** MCPyV viral transcripts were measured via RT-qPCR in primary adult human dermal fibroblast (aHDF) and **(B)** BJ-hTERT cells cultured as 2D monolayers or 3D spheroids using magnetic levitation after 4 days. 3D microenvironment greatly enhanced both wild-type (WT) and E3 mutant virus (E3(-)) early/late gene transcription compared to 2D monolayer culture. RNAse P was used as a reference gene to normalize transcripts. n = 3. Unpaired multiple Student’s *t* test using the Holm-Sidak method were used to test statistical significance and error bars are reported as standard error of the mean (SEM).

**S2 Fig.** **Detection of MCPyV viral protein expression in viral supernatant-infected nHDF cells.** **(A)** Detection of MCPyV DNA from viral supernatants isolated from empty vector- or MCPyV-transfected nHDF cells cultured as a monolayer or as a spheroid (n = 3). Statistical significance was analyzed using a one-way analysis of variance (ANOVA) with Dunnett’s multiple-comparison tests. **(B)** Immunofluorescent detection of LT (red) and VP1 (green) in monolayer nHDF cells infected with mock or viral supernatants supplemented with polybrene (12 µg/mL) at 4 days post-infection. Arrows denote positive for LT and/or VP1 staining. **(C)** Detection of LT expression from MCPyV-infected nHDF spheroids by immunoprecipitation (IP) coupled with immunoblotting with CM2B4 antibody. HEK293 cell lysates transfected with MCPyV LT were used as positive controls. * Denotes non-specific bands.

**S3 Fig. MCPyV virions are associated with extracellular vesicles.** Negative TEM staining of the MCPyV-EVs. The pellet from supernatants of nHDF-transfected with MCPyV genome was isolated by centrifugation (10,000 x g) and observed by TEM analysis. **(A)** MCPyV virions associated with EVs or **(B)** naked MCPyV virus particles were detected. Various virions were measured in size. Scale bar = 100 nm.

**
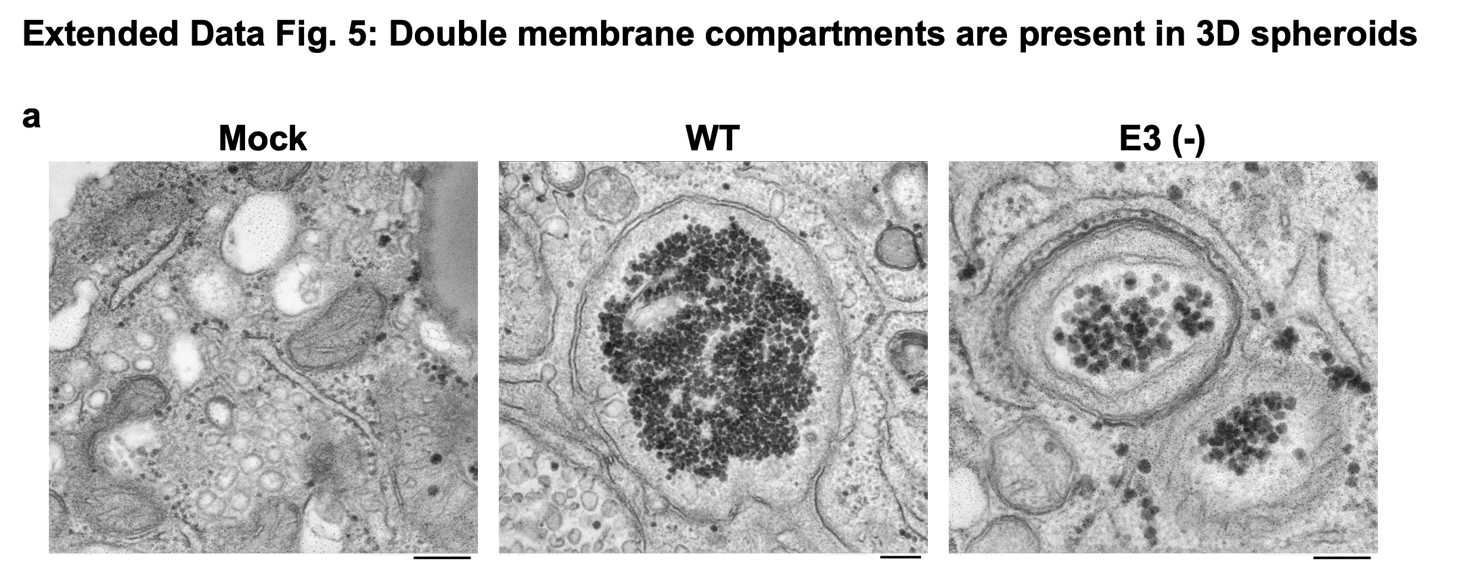
**

**S4 Fig. Double membrane compartments containing MCPyV virions are present in MCPyV-infected nHDF cell spheroids.** Dense MCPyV virus-like particles largely accumulated inside of double membraned-endosomal compartments are observed in cells infected with MCPyV. nHDF cells infected with MCPyV for 4 days in 3D culture were harvested and fixed. Thin sections (70 nm) of spheroids (one third of the distance from the top) were stained with uranyl acetate and lead citrate and imaged at 60 kV using a JEOL JEM1400 Transmission Electron Microscope (JEOL USA Inc.). Scale bar = 200 nm.

**S5 Fig. MCPyV capsid antibodies neutralize both EV-associated and EV-free MCPyV infection.** nHDF spheroids were incubated with mouse IgG or α-MCPyV capsid antibodies (9B2 or G6) and then infected with MCPyV extracellular vesicle-depleted (EV(-), left) or extracellular vesicle-enriched (EV(+), right) media for 1 day. Statistical significance was analyzed using a one-way analysis of variance (ANOVA) with Dunnett’s multiple-comparison tests.

**S6 Fig. Pathway enrichment analysis of MCPyV- and 3D spheroids-associated DEGs. (A)** Pathway enrichment of downregulated (left, >0.5-fold) and upregulated pathways (right, >4-fold) comparing MCPyV- vs mock-infected 3D spheroids. **(B)** Many of the enriched pathways with MCPyV infection in nHDF spheroids (3D) compared to monolayers (2D) were related to the “epithelial cell differentiation”, “actin filament organization”, “Wnt signaling/planar cell polarity”, and “morphogenesis of epithelium”.


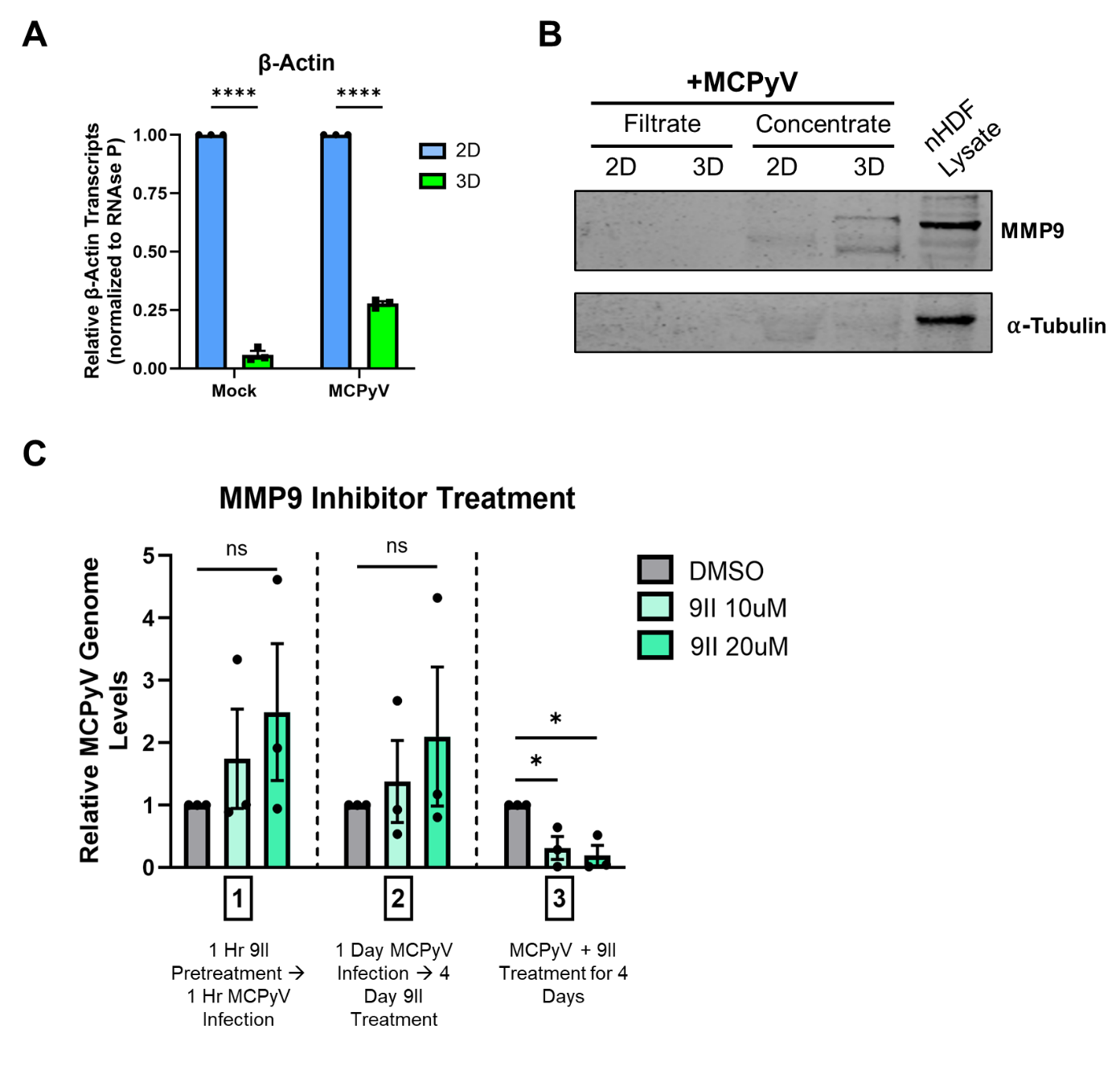


**S7 Fig. MMP9 and actin regulation in the 3D microenvironment. (A)** Decreased β-Actin expression was 3D microenvironment-dependent. RT-qPCR analysis of β-Actin transcript levels from mock- or MCPyV-infected spheroids 4 days post-infection (n = 3). RNAse P was used as a housekeeping gene. **(B)** Immunoblot analysis of secreted MMP9. Conditioned media from MCPyV-transfected monolayers or spheroids were harvested and concentrated using a 30 kDa cutoff centrifugal filter at 4 days post-transfection (n = 2). Filtrate and concentrate loading were normalized to cell count and were analyzed via immunoblot and probed for MMP9. Increased secretion of MMP9 was observed from 3D compared to 2D conditioned media. Lysates from monolayer nHDF cells served as positive control for MMP9 detection. α-tubulin was used as an intracellular protein control. **(C)** MMP9 activation increases MCPyV infection in nHDF 3D culture. MMP9 inhibitor treatment (9II) inhibited MCPyV infection in 3D (n = 3).

**References**

1. Pham AM, Ortiz LE, Lukacher AE, Kwun HJ. Merkel Cell Polyomavirus Large T Antigen Induces Cellular Senescence for Host Growth Arrest and Viral Genome Persistence through Its Unique Domain. Cells. 2023;12(3). Epub 20230120. doi: 10.3390/cells12030380. PubMed PMID: 36766726; PubMed Central PMCID: PMCPMC9913222.

2. Kwun HJ, Chang Y, Moore PS. Protein-mediated viral latency is a novel mechanism for Merkel cell polyomavirus persistence. Proc Natl Acad Sci U S A. 2017;114(20):E4040-E7. Epub 20170501. doi: 10.1073/pnas.1703879114. PubMed PMID: 28461484; PubMed Central PMCID: PMCPMC5441811.

3. Pham AM, Kwun HJ. Casein kinase 1α mediates phosphorylation of the Merkel cell polyomavirus large T antigen for β-TrCP destruction complex interaction and subsequent degradation. mBio. 2024:e0111724. Epub 20240628. doi: 10.1128/mbio.01117-24. PubMed PMID: 38940554.

4. Altan-Bonnet N, Perales C, Domingo E. Extracellular vesicles: Vehicles of en bloc viral transmission. Virus Res. 2019;265:143-9. Epub 20190327. doi: 10.1016/j.virusres.2019.03.023. PubMed PMID: 30928427.

5. Théry C, Witwer KW, Aikawa E, Alcaraz MJ, Anderson JD, Andriantsitohaina R, et al. Minimal information for studies of extracellular vesicles 2018 (MISEV2018): a position statement of the International Society for Extracellular Vesicles and update of the MISEV2014 guidelines. J Extracell Vesicles. 2018;7(1):1535750. Epub 20181123. doi: 10.1080/20013078.2018.1535750. PubMed PMID: 30637094; PubMed Central PMCID: PMCPMC6322352.

6. Wubbolts R, Leckie RS, Veenhuizen PT, Schwarzmann G, Möbius W, Hoernschemeyer J, et al. Proteomic and biochemical analyses of human B cell-derived exosomes. Potential implications for their function and multivesicular body formation. J Biol Chem. 2003;278(13):10963-72. Epub 20030107. doi: 10.1074/jbc.M207550200. PubMed PMID: 12519789.

7. Feinstone SM, Mihalik KB, Kamimura T, Alter HJ, London WT, Purcell RH. Inactivation of hepatitis B virus and non-A, non-B hepatitis by chloroform. Infect Immun. 1983;41(2):816-21. doi: 10.1128/iai.41.2.816-821.1983. PubMed PMID: 6409813; PubMed Central PMCID: PMCPMC264712.

8. Handala L, Blanchard E, Raynal PI, Roingeard P, Morel V, Descamps V, et al. BK Polyomavirus Hijacks Extracellular Vesicles for. J Virol. 2020;94(6). Epub 20200228. doi: 10.1128/JVI.01834-19. PubMed PMID: 31896595; PubMed Central PMCID: PMCPMC7158717.

9. Morris-Love J, O'Hara BA, Gee GV, Dugan AS, O'Rourke RS, Armstead BE, et al. Biogenesis of JC polyomavirus associated extracellular vesicles. J Extracell Biol. 2022;1(5). Epub 20220403. doi: 10.1002/jex2.43. PubMed PMID: 36688929; PubMed Central PMCID: PMCPMC9854252.

10. Nwogu N, Ortiz LE, Whitehouse A, Kwun HJ. Merkel Cell Polyomavirus Small Tumor Antigen Activates Matrix Metallopeptidase-9 Gene Expression for Cell Migration and Invasion. J Virol. 2020;94(19). Epub 2020/09/15. doi: 10.1128/JVI.00786-20. PubMed PMID: 32669331; PubMed Central PMCID: PMCPMC7495391.

11. Ge SX, Jung D, Yao R. ShinyGO: a graphical gene-set enrichment tool for animals and plants. Bioinformatics. 2020;36(8):2628-9. doi: 10.1093/bioinformatics/btz931. PubMed PMID: 31882993; PubMed Central PMCID: PMCPMC7178415.

12. Ge SX, Son EW, Yao R. iDEP: an integrated web application for differential expression and pathway analysis of RNA-Seq data. BMC Bioinformatics. 2018;19(1):534. Epub 20181219. doi: 10.1186/s12859-018-2486-6. PubMed PMID: 30567491; PubMed Central PMCID: PMCPMC6299935.

S1 Table. Mass spectrometric analysis of MCPyV-EVs.

S2 Table. RNA-seq analysis of MCPyV-EV infected nHDF cells.

S3 Table. Integrated differential expression and pathway analysis of RNA-seq data.

S4 Table. Antibodies used in this study.

S5 Table. Primers used in this study.
