# Supplementary material for: Merkel cell polyomavirus exploits extracellular vesicles for skin infection and host immune evasion through activated Wnt signaling
Source: PLoS Pathog. 2026 Jun 23;22(6):e1014360. doi: 10.1371/journal.ppat.1014360 (PMC13289936; doi:10.1371/journal.ppat.1014360)
Supplement: Attachment — Submitted filename: Response to Reviewers.docx [file ppat.1014360.s008.docx]

Dear Editor:

We are grateful to the reviewers for taking the time to read our study and for providing helpful and constructive comments that allowed us to improve our original manuscript. All comments from the reviewers were thoroughly addressed as described below.

Point-by-point Response to Reviews:

**Reviewer #1:**

**Major Revisions**

**Reviewer #1:** In Figure 4D what is responsible for the 25% of infectivity that remains after EV depletion. Is this due to virions not associated with EV? Would anti-MCPyV at this stage inhibit further? This would be a good control to show that both EV associated and non-associated virus are at play.

**Reply:** We repeated our experiments for the EV depletion and included it in Fig. 4D. Our TEM analysis showed a subset of naked MCPyV virion remained in the supernatant; however, infectivity of naked MCPyV virion was clearly limited compared to the EV-associated MCPyV virions (Fig. 4D). In addition, MCPyV supernatant (MCPyV-EVs) was treated with chloroform to dissolve EV membranes, effectively lysing EVs in Fig. 4F. Chloroform or proteinase K treatment reduced infectivity and treatment of both completely impaired MCPyV infection, indicating that both EV-associated and non-associated viruses are at play. In S5 Fig, we included additional neutralization assay showing both EV-associated (EV(+)) and EV free (EV(-)) are inhibited by MCPyV capsid antibodies.

**Reviewer #1:** In Figure 6E they show that MMP9 inhibition reduces infection. Is the effect at the level of binding or entry of virus as MMP9 inhibition would be expected to reduce extracellular matrix remodeling and possibly access to the cell surface.

**Reply:** We thank the reviewer for the suggestion. To examine how MMP9 might affect MCPyV infection, we preincubated spheroids with MMP9 inhibitor 9II for one hour and then infected the spheroids for one hour to examine viral entry. We found that 9II had no effect on MCPyV viral entry. We also examined the effect of 9II on post-entry replication by treating 9II for 4 days at 1 day post-infection. We also observed no effect on virus infection by MMP9 inhibition with this condition. Only when cells were infected and treated with 9II at the same time before spheroid formation, we did observe a reduction in infectivity. Thus, we believe that MMP9 is required to break down the ECM microenvironment inside of the spheroid and this allows for enhanced viral infection and transmission. The new data are included in S7C Fig and Fig. 6E. Lines 323-330 (unmarked manuscript) also state this information.

**Minor Revisions**

**Reviewer #1: (No Response)**

**Reviewer #2:**

**Major Revisions**

**Reviewer #2:**  The quantification of the data between the 3D vs 2D system is confusing. Infection data from 3D vs. 2D models should be normalized to input virus (or is the input virus due to transfection of the viral genome?). The authors cannot accurately claim the 3D model is more infectable than the 2D model if no effort or control is made to ensure an equal amount of virus is initially used across conditions. How does a 3D system better support viral genome replication and maintenance when compared to a 2D system?

**Reply:** We had reported in our original manuscript that we used 2 x 10^8^ MCPyV genome copies to infect both 2D or 3D cultures in the methods section under **MCPyV Infection and MCPyV Genome Analysis**, and **Antibody Neutralization and Heparin Assays**. Equal amounts of virus to infect 3D and 2D were consistently used throughout all experiments. We also seeded equal cell numbers for all experiments. We have updated the methods section to note that we infected 2D or 3D cultures with 2 x 10^8^ or 2 x 10^9^ MCPyV genome copies. All figure legends have also been updated to report the infectious dose used for each experiment. All qPCR data were generated using the same amount of DNA template with housekeeping gene analysis as a control, which was a practice to compare between 2D and 3D samples. In addition, our data suggest that 3D culture supports enhanced MCPyV genome replication and persistence compared to 2D culture due to activated Wnt/MMP signaling and the wound healing environment in 3D microenvironment which will be further addressed in the below comments.

**Reviewer #2:** Measuring viral genome via qPCR is not a rigorous measurement of infection as this measurement is directly confounded by the input virus’ DNA. Western blot for T-antigen or RT-qPCR against gene targets is necessary to detect infection (in addition to the proper controls above).

**Reply:** We have updated the manuscript to include RT-qPCR in Fig. 3F and MCPyV LT expression analysis in S2 Fig. As expected, MCPyV LT transcripts are increased by ~4-fold in 3D spheroids compared to 2D monolayers (Fig. 3F, lines 181-184). Because LT protein expression in MCPyV infected cells was not enriched enough to be detected by a direct immunoblotting due to the dilution of fibroblast lysates, it was necessary to **concentrate the sample.** This is not due to low infectivity but rather caused by low expression regulated by LT-mediated early gene transcription inhibition (PMID: [32306957](https://pubmed.ncbi.nlm.nih.gov/32306957/)), downregulation of early gene transcription by viral miRNAs (PLoS Pathog 11(7): e1004974), and rapid LT protein turnover-mediated by cellular E3 ligases (PMID: [28461484](https://pubmed.ncbi.nlm.nih.gov/28461484/)), which are the part of mechanisms known for virus persistence. Thus, we conducted immunoprecipitation to pull down and detect MCPyV LT in MCPyV-infected spheroids seen in S2C Fig (Lines 188-190). We also would like to point out that we already had shown the detection of MCPyV proteins (LT and VP1) via immunofluorescence in our original manuscript (Figures 3G and S2B).

**Reviewer #2:** In Fig. 4D, how does a supernatant with EV-containing virus display higher infection than an EV-free supernatant. Does this suggest that virus entry leading to infection is more efficient when the virus is in an EV? How does this work? Also, in Fig. 4G, how does heparin block ER-associated virus?

**Reply:** Yes, our data suggests that MCPyV-EVs are more efficient for infection compared to naked virions/EV-free media. We changed Fig. 4D (Lines 210-216) to better reflect this data. When fibroblasts were infected with EV enriched (EV+) media, MCPyV infection efficiency is increased by ~7.5 fold compared to EV-depleted (EV-) media. EVs can package numerous virions at once, which we observed in Fig. 4A, and thus can increase the multiplicity of infection leading to a more robust infection. This is discussed in lines 387-391. EVs can also utilize different entry receptors or mechanisms than naked viruses and therefore could be taken up more efficiently depending on the uptake receptor/mechanism. This will require future studies to discern differences in entry/uptake between naked and EV-associated MCPyV virions. EVs can also utilize heparan sulfate proteoglycans (HPSGs) for cell surface attachment and uptake (PMID: [25143819](https://pubmed.ncbi.nlm.nih.gov/25143819/)) similar to naked MCPyV virions. Therefore, treating cells with heparin can bind to HSPGs and block attachment of both naked viruses and EV-associated viruses. This was discussed in lines 238-242.

**Reviewer #2:**  In Fig. 5, there is not sufficient evidence to support that activated Wnt-signalling promotes MCPyV infection. The authors do not find elevated MMP9 protein levels via Western blot in the 3D culture despite robustly elevated transcript levels. Additionally, inhibitor 1 against MMP9 had no effect on infection, while inhibitor 2 only decreased infection modestly. Why inhibitor 2 modestly decreases infection when targeting the active form of MMP9, which is robustly decreased via Western blot, is not clear. Overall, the proteomics data do not strongly support virus infection creating an immunosuppressive environment that allows the virus to evade immune detection.

**Reply:** We found that intracellular active MMP9 was decreased in 3D spheroids, suggesting its secretion. In our original manuscript, this was explained. MMP9 often shows low detectable expression inside of the cells due to its rapid secretion into the extracellular environment once activated. We have included the western blot detection of secreted MMP9 from monolayer or spheroid conditioned media (now S7B Fig, Lines 322-323). We found that MMP9 was increased in the conditioned media compared to monolayer conditioned media. MMP9 **Inhibitor 9II is considered the more potent compound than inhibitor 9I**. Thus, we have repeated our experiment examining the role of MMP9 in viral entry, post-entry replication, and intra-spheroid ECM using MMP9 inhibitor 9II (S6E Fig and S7C Fig, Lines 327-334). See above comments.

We had shown in our original manuscript that ISGs, IFITM3, MIF, Galectins were downregulated in MCPyV-EV cargo, which points to a significant suppression of immune and inflammatory responses. We have additionally included the expression of TGFBR1, 2, 3 to the mass-spec analysis of MCPyV-EV cargo loading in Fig. 5F. Our data analysis showed that TGFBR1 and TGFBR2 were upregulated while TGFBR3 is downregulated in MCPyV-EVs. These receptors are essential for TGF-beta signaling and inducing immunosuppression (PMID: 30995507). TGFBR3 can act as a negative regulator of TGF-beta signaling by sequestering TGF-beta and preventing its binding to TGFBR2 (PMID: 8106553). These changes in TGF-beta signaling are essential for creating an immunosuppressive environment by promoting Treg differentiation and proliferation, while inhibiting dendritic cell and macrophage function. We included our discussion with references in lines 281-289.

**Reviewer #2:**  Also, the RNAseq data in Fig. 6 is very descriptive and the connection between activated Wnt signaling in the 3D system and enhanced virus replication is rather weak and not strongly supported.

**Reply:** We disagree with this comment. We analyzed specific signaling pathways-related genes individually and confirmed some of our RNA-seq data in Fig.6. We also have analyzed our data and listed genes and pathway analysis in our Supplementary data (S2 and S3 Tables). We have now included additional pathway analysis in S6 Fig. Our pathway analysis clearly shows Wnt signaling pathway activation in 3D, which is also consistent with the previous observation shown from Dr. You’s group’s study (PMID: [27212661](https://pubmed.ncbi.nlm.nih.gov/27212661/)) as we mentioned in the manuscript. Lastly, we confirmed increases in both MMP9 transcript expression (Fig. 6C) and MMP9 secretion (S7B Fig) in 3D spheroids.

**Minor Revisions**

**Reviewer #2**: The authors are consistently unclear when either RT-qPCR or qPCR was used throughout the text, figure captions, and methods, thus making interpretations convoluted. Additionally, how data are processed and normalized is not thoroughly described in the figure captions. The authors should also indicate n.s. on bar charts where the data are not significant.

**Reply:** We thank the reviewer for this suggestion. RT-qPCR or qPCR data were added to figure legends when appropriate. Figures were also additionally labeled with RT-qPCR or relative transcript levels to aid with clarity and distinguishment between the two experiments. RNAse P was denoted as a control housekeeping gene for normalization for RT-qPCR in all figure legends and is described in the methods section as well. ns was added to figures when appropriate.

**Reviewer #2:**  Fig. 3B: The method and technique used to measure MCPyV infection is confusing. The authors note in Fig. 3A that an increased copy number from spheroid cell culture was obtained; when these fractions are pooled, this increased copy number will remain. Observing an increased infection rate in Fig. 3B could be due to a higher copy number at the start of infection and not due to a physiological advantage from the 3D system. Additionally, measuring the genome copy number is an insufficient manner to measure infection because this again is confounded by input genome. The authors should normalize the copy number for the infection and measure a marker to better track infection (i.e., RT-qPCR for genome, Western blot for T-antigen).

**Reply:** Fig. 3B is a confirmation of Fig. 3A data by testing MCPyV infection isolated from the pool of fractions with high copy number of MCPyV. We have revised our Fig. 3B and legend to clarify the results. Experiments for LT transcripts levels (Fig. 3F) and LT protein detection data (S2C Fig) were added.

**Reviewer #2:**  Fig. 3C: Inclusion of a 2D culture as a control is necessary to interpret the levels from a 3D culture.

**Reply:** We have recorded MCPyV viral titers from 2D and 3D cultures in S2A Fig. The production of MCPyV virus was significantly reduced from 2D (~50 pg/uL) compared to 3D cultures (~100-300 pg/uL).

**Reviewer #2:** Fig. 3E: Similar to Fig. 3B, normalization of the input virus is required to interpret efficiency of infectivity. And again, a better measurement of infection is required.

**Reply:** Experiments for LT transcripts levels (Fig. 3F) and LT protein detection data (S2C Fig) were added.

**Reviewer #2:** Fig. 4D: There is no control to indicate that the same copy number or titer of MCPyV was used for infection in the +EV or -EV conditions. It’s unclear whether the additional steps to generate EV-free virus reduced the copy number and thus leads to decreased infection.

**Reply:** We have updated Fig. 4D and included that we infected spheroids with 2 x 10^9^ MCPyV genome copies using either EV+ or EV- media in the figure legend.

**Reviewer #2:** Fig. 4E: It’s unclear why the overwhelming majority of detected MCPyV genome is free of capsid and sensitive to DNase. The authors should combine Figs. 4E and 4F; MCPyV DNA within EVs should be resistant to DNase treatment when either proteinase K or chloroform is used independently, but sensitive when combined.

**Reply:** We have revised Fig. 4F to include viral supernatants treated with chloroform, proteinase K, or both that were used for infections (Lines 220-234). Spheroids were infected using 2 x 10^9^ genome copies using all the treated supernatants. Chloroform treatment, which will solubilize EV membranes, led to a decreased infection with corroborates the data from 4D, demonstrating the higher efficiency of MCPyV-EVs compared to naked viruses. Proteinase K treatment also reduced MCPyV infection, likely by degraded naked viruses, but leaving MCPyV-EVs intact for infection. Proteinase K treatment likely also degraded MCPyV virions tethered to the outside of EVs and therefore would not be protected. Combined treatment of chloroform+proteinase K treatment led to a complete abrogation of MCPyV infection. These data suggest that encapsidated virus particles are packaged inside of EVs. Free MCPyV DNA might come from dead cells at the necrotic core of spheroids releasing DNA into the supernatant.

**Reviewer #2:** Fig. 4G: Text says that heparin is required for EV-associated MCPyV and naked MCPyV but the figure makes no distinction between the subset of MCPyV used.

**Reply:** Viral supernatants containing both EVs and naked virions were used for heparin pretreatment infections. These data suggest that heparin can inhibit both MCPyV-EVs and naked virions as MCPyV infection efficiency was largely reduced.

**Reviewer #2:** Fig. 5A: again, a more robust measurement of infection is required. Additionally, given that infection is blocked by roughly half, writing that “EV membranes can protect virions against neutralizing antibodies” is not valid. This assay should be redone with +EV or EV-free media (with a normalized copy number of MCPyV).

**Reply:** We conducted a neutralization assay by incubating anti-G6 (VLP) and 9B2 antibodies with EV(+) and EV-free (EV(-)) media (S5 Fig, Lines 253-257).

**Reviewer #2:** Text at the end of Fig. 5 “These data suggest MCPyV-infected cells could enhance the susceptibility of infection by inducing an immunosuppressive environment that allows pathogens to avoid immune detection and facilitates the spread of infection to neighboring cells.” is not explicitly supported by the data and should be removed or moved to the discussion.

**Reply:** Additional mass spec data regarding TGF-beta receptors was included in Fig. 5F as mentioned above. TGF-beta signaling is highly responsible for developing an immunosuppressive environment by dampening the immune response. We also included more analysis on MCPyV-dependent RNA-Seq pathway enrichment in S6A Fig, comparing MCPyV-3D and Mock-3D (Lines 298-301). Pathways downregulated by MCPyV include immune response signaling pathways such as “JAK-STAT signaling” and “MAPK signaling”. These additional data further suggest the ability of MCPyV to modulate the host immunity. The text “These data suggest MCPyV-infected cells could enhance the susceptibility of infection by inducing an immunosuppressive environment that allows pathogens to avoid immune detection and facilitates the spread of virions to neighboring cells” was moved to the discussion section in Lines 403-406.

**Reviewer #2:** Fig. 6B: The overwhelming factor driving differential expression of downstream Wnt signaling proteins is from the 2D vs. 3D cell culture model. It’s unclear whether MCPyV addition affects this at all. Analysis from the mock 2D vs. 3D models should also be included to determine the relevance of MCPyV infection on these genes.

**Reply:** In our previous version of the manuscript, we had explained that Wnt signaling proteins are modulated by 3D cell culture, which could enhance MCPyV infection. For clarity, we additionally confirmed that β-actin transcript levels were downregulated in both mock- and MCPyV-infected spheroids compared to monolayers (S7A Fig). These data demonstrate that changes in pathways involved in wound healing are mainly 3D-dependent. Our data conclude that 3D environments are critical for accurately modeling the complex signaling pathways in wound healing, as they allow cells to interact with a matrix and each other, a process not possible in 2D monolayers.

**Reviewer #2:** Fig. 6C+D: 6C finds elevated MMP9 transcripts yet Fig. 6D finds a decrease in active MMP9 protein and a nearly equivalent level of MMP9 pro-form, both of which are in conflict with the elevated transcript finds from Fig. 6C.

**Reply:** We have added the updated data in S7B Fig to display secreted levels of MMP9 from 2D and 3D conditioned media as mentioned above. Both pro- and active-forms of MMP9 were increased in 3D conditioned media compared to 2D conditioned media.

**Reviewer #2:** Fig. 6E: A more robust measurement of infection is required. Also the text stating “reaffirming that Wnt MMP signaling may play a critical role in regulating the MCPyV lifecycle” is unfounded considering inhibitor 1 had no effect on MCPyV infection. Additionally, it is unclear whether the infection was done in a 2D or 3D model and is not described adequately in the text or figure captions. If this infection was done in the 3D model, it is unclear why an inhibitor against the active MMP9 would decrease infection given that the active form was found to be robustly decreased in the 3D model.

**Reply:** We have updated the figure legend to denote that the infection and MMP9 inhibitor treatment was conducted on 3D spheroids. As mentioned above, we have included figures that demonstrate increased MCPyV early gene transcripts (Fig. 3F) and secreted MMP9 expression (S7B Fig) in 3D spheroid compared to monolayer cultures. MMP9 **Inhibitor 9II is considered the more potent compound than inhibitor 9I**. Thus, we have repeated our experiment and Fig. 6E was updated as we mentioned above.
