# Supplementary material for: Merkel cell polyomavirus exploits extracellular vesicles for skin infection and host immune evasion through activated Wnt signaling
Source: PLoS Pathog. 2026 Jun 23;22(6):e1014360. doi: 10.1371/journal.ppat.1014360 (PMC13289936; doi:10.1371/journal.ppat.1014360)
Supplement: Peer review history

# Peer review: Decision Letter 0

**DOI:** 10.1371/journal.ppat.1014360.r001

**Authors:** Blossom Damania, Paul Lambert

5 Apr 2026

PPATHOGENS-D-26-00663

Merkel Cell Polyomavirus Exploits Extracellular Vesicles for Skin Infection and Host Immune Evasion Through Activated Wnt Signaling

PLOS Pathogens

Dear Dr. Kwun,

Thank you for submitting your manuscript to PLOS Pathogens. After careful consideration, we feel that it has merit but does not fully meet PLOS Pathogens's publication criteria as it currently stands. Therefore, we invite you to submit a revised version of the manuscript that addresses the points raised during the review process.

Please submit your revised manuscript by Jun 04 2026 11:59PM. If you will need more time than this to complete your revisions, please reply to this message or contact the journal office at plospathogens@plos.org. When you're ready to submit your revision, log on to https://www.editorialmanager.com/ppathogens/ and select the 'Submissions Needing Revision' folder to locate your manuscript file.

Please include the following items when submitting your revised manuscript:

* A letter that responds to each point raised by the editor and reviewer(s). You should upload this letter as a separate file labeled 'Response to Reviewers'. This file does not need to include responses to any formatting updates and technical items listed in the 'Journal Requirements' section below.

* A marked-up copy of your manuscript that highlights changes made to the original version. You should upload this as a separate file labeled 'Revised Manuscript with Track Changes'.

* An unmarked version of your revised paper without tracked changes. You should upload this as a separate file labeled 'Manuscript'.

If you would like to make changes to your financial disclosure, competing interests statement, or data availability statement, please make these updates within the submission form at the time of resubmission. Guidelines for resubmitting your figure files are available below the reviewer comments at the end of this letter.

We look forward to receiving your revised manuscript.

Kind regards,

Paul F. Lambert

Academic Editor

PLOS Pathogens

Blossom Damania

Section Editor

PLOS Pathogens

Sumita Bhaduri-McIntosh

Editor-in-Chief

PLOS Pathogens

orcid.org/0000-0003-2946-9497

Michael Malim

Editor-in-Chief

PLOS Pathogens

orcid.org/0000-0002-7699-2064

**Journal Requirements:**

If the reviewer comments include a recommendation to cite specific previously published works, please review and evaluate these publications to determine whether they are relevant and should be cited. There is no requirement to cite these works unless the editor has indicated otherwise.

1) Please provide an Author Summary. This should appear in your manuscript between the Abstract (if applicable) and the Introduction, and should be 150-200 words long. The aim should be to make your findings accessible to a wide audience that includes both scientists and non-scientists. Sample summaries can be found on our website under Submission Guidelines:

https://journals.plos.org/plospathogens/s/submission-guidelines#loc-parts-of-a-submission

2) Your manuscript is missing the following sections: Introduction.  Please ensure all required sections are present and in the correct order. Make sure section heading levels are clearly indicated in the manuscript text, and limit sub-sections to 3 heading levels. An outline of the required sections can be consulted in our submission guidelines here:

https://journals.plos.org/plospathogens/s/submission-guidelines#loc-parts-of-a-submission

3) Please upload all main figures as separate Figure files in .tif or .eps format. For more information about how to convert and format your figure files please see our guidelines:

https://journals.plos.org/plospathogens/s/figures

4) In the online submission form, you indicated that your data will be submitted to a repository upon acceptance. We strongly recommend all authors deposit their data before acceptance, as the process can be lengthy and hold up publication timelines. Please note that, though access restrictions are acceptable now, your entire minimal dataset will need to be made freely accessible if your manuscript is accepted for publication. This policy applies to all data except where public deposition would breach compliance with the protocol approved by your research ethics board. If you are unable to adhere to our open data policy, please kindly revise your statement to explain your reasoning and we will seek the editor's input on an exemption.

5) Please amend your detailed Financial Disclosure statement. This is published with the article. It must therefore be completed in full sentences and contain the exact wording you wish to be published.

1) State what role the funders took in the study. If the funders had no role in your study, please state: "The funders had no role in study design, data collection and analysis, decision to publish, or preparation of the manuscript."

2) If any authors received a salary from any of your funders, please state which authors and which funders..

If you did not receive any funding for this study, please simply state: u201cThe authors received no specific funding for this work.u201d

6) Some material included in your submission may be copyrighted. According to PLOS’s copyright policy, authors who use figures or other material (e.g., graphics, clipart, maps) from another author or copyright holder must demonstrate or obtain permission to publish this material under the Creative Commons Attribution 4.0 International (CC BY 4.0) License used by PLOS journals. Please closely review the details of PLOS’s copyright requirements here: PLOS Licenses and Copyright. If you need to request permissions from a copyright holder, you may use PLOS's Copyright Content Permission form.

Please respond directly to this email and provide any known details concerning your material's license terms and permissions required for reuse, even if you have not yet obtained copyright permissions or are unsure of your material's copyright compatibility. Once you have responded and addressed all other outstanding technical requirements, you may resubmit your manuscript within Editorial Manager.

Potential Copyright Issues:

i) We note that Figure 7 is created through BioRender. Please confirm that you hold a Premium account and provide a pdf copy of the CC BY 4.0 Licence as provided by BioRender. For instructions on how to generate a CC BY 4.0 license for your figure, please see the guidelines here: https://help.biorender.com/hc/en-gb/articles/21282341238045-Publishing-in-open-access-resources

Please ensure the figure legend contains appropriate attribution text & a link to biorender.com

If you are using the free assets from BioRender, we are unable to publish these images as they are licenced under a stricter licence than CC BY 4.0. In this case we ask you to remove the BioRender images and replace them with open source alternatives.

See these open source resources you may use to replace images / clip-art:

- https://bioart.niaid.nih.gov/

- https://bioicons.com/

- https://healthicons.org/

- https://scidraw.io/

- https://reactome.org/icon-lib

- https://www.phylopic.org/images

- https://journals.plos.org/plosbiology/article?id=10.1371/journal.pbio.3002395

**Reviewers' Comments:**

Reviewer's Responses to Questions

**Part I - Summary**

Please use this section to discuss strengths/weaknesses of study, novelty/significance, general execution and scholarship.

Reviewer #1: The article by Pham and colleagues “Merkel Cell Polyomavirus Exploits Extracellular Vesicles for Skin Infection and Host Immune Evasion Through Activated Wnt Signaling” is a major breakthrough in MCPyV biology. This has been a difficult virus to propagate but the authors here show convincingly that 3D cultures of skin dermal fibroblasts support robust infection and progeny virus release vs monolayers. They used three different methods to generate the 3D cultures and all gave similar results. This system was then completely characterized and they found that a significant fraction of infectious virus was contained with the extracellular vesicle fraction in density gradients. The EV were fully characterized and shown to contain infectious virus. Finally, they show that in the 3D format Wnt signaling resembling the wound healing process was activated and promoted enhanced replication of the virus. Inhibition of MMP9 reduced virus replication suggesting thar extracellular matrix remodeling may play a role in infectious spread of MCPyV either as free virions or as EV associated virions. This was a well-controlled study that establishes a new robust system for the exploration of MCPyV biology.

Reviewer #2: In the manuscript by Pham et al., the authors investigate whether a 3D spheroid system derived from primary human dermal fibroblasts more faithfully recapitulates native MCPyV infection compared to the 2D cell culture models that are typically used. The authors find that three independent 3D models increase infection compared to 2D models, in a manner recapitulated across multiple cell lines. The authors find that in addition to enabling infection, the 3D model also produces infectious virions in the supernatant following infection. By electron microscopy, the authors find the supernatant virus is associated with extracellular vesicles, which are found to be important for infection. Finally, the authors find increased expression of Wnt-signalling proteins and find this pathway may promote MCPyV infection. Despite these findings, the current manuscript lacks many necessary controls and the quality of the data are not sufficiently high to justify publication in this journal.

**********

**Part II – Major Issues: Key Experiments Required for Acceptance**

Please use this section to detail the key new experiments or modifications of existing experiments that should be absolutely required to validate study conclusions.

Generally, there should be no more than 3 such required experiments or major modifications for a "Major Revision" recommendation. If more than 3 experiments are necessary to validate the study conclusions, then you are encouraged to recommend "Reject".

Reviewer #1: In Figure 4D what is responsible for the 25% of infectivity that remains after EV depletion. Is this due to virions not associated with EV? Would anti-MCPyV at this stage inhibit further? This would be a good control to show that both EV associated and non-associated virus are at play.

In Figure 6E they show that MMP9 inhibition reduces infection. Is the effect at the level of binding or entry of virus as MMP9 inhibition would be expected to reduce extracellular matrix remodeling and possibly access to the cell surface.

Reviewer #2: 1. The quantification of the data between the 3D vs 2D system is confusing. Infection data from 3D vs. 2D models should be normalized to input virus (or is the input virus due to transfection of the viral genome?). The authors cannot accurately claim the 3D model is more infectable than the 2D model if no effort or control is made to ensure an equal amount of virus is initially used across conditions. How does a 3D system better support viral genome replication and maintenance when compared to a 2D system?

Measuring viral genome via qPCR is not a rigorous measurement of infection as this measurement is directly confounded by the input virus’ DNA. Western blot for T-antigen or RT-qPCR against gene targets is necessary to detect infection (in addition to the proper controls above).

2. In Fig. 4D, how does a supernatant with EV-containing virus display higher infection than an EV-free supernatant. Does this suggest that virus entry leading to infection is more efficient when the virus is in an EV? How does this work? Also, in Fig. 4G, how does heparin block ER-associated virus?

3. In Fig. 5, there is not sufficient evidence to support that activated Wnt-signalling promotes MCPyV infection. The authors do not find elevated MMP9 protein levels via Western blot in the 3D culture despite robustly elevated transcript levels. Additionally, inhibitor 1 against MMP9 had no effect on infection, while inhibitor 2 only decreased infection modestly. Why inhibitor 2 modestly decreases infection when targeting the active form of MMP9, which is robustly decreased via Western blot, is not clear. Overall, the proteomics data do not strongly support virus infection creating an immunosuppressive environment that allows the virus to evade immune detection.

Also, the RNAseq data in Fig. 6 is very descriptive and the connection between activated Wnt signaling in the 3D system and enhanced virus replication is rather weak and not strongly supported.

**********

**Part III – Minor Issues: Editorial and Data Presentation Modifications**

Please use this section for editorial suggestions as well as relatively minor modifications of existing data that would enhance clarity.

Reviewer #1: (No Response)

Reviewer #2: The authors are consistently unclear when either RT-qPCR or qPCR was used throughout the text, figure captions, and methods, thus making interpretations convoluted. Additionally, how data are processed and normalized is not thoroughly described in the figure captions. The authors should also indicate n.s. on bar charts where the data are not significant.

Fig. 3B: The method and technique used to measure MCPyV infection is confusing. The authors note in Fig. 3A that an increased copy number from spheroid cell culture was obtained; when these fractions are pooled, this increased copy number will remain. Observing an increased infection rate in Fig. 3B could be due to a higher copy number at the start of infection and not due to a physiological advantage from the 3D system. Additionally, measuring the genome copy number is an insufficient manner to measure infection because this again is confounded by input genome. The authors should normalize the copy number for the infection and measure a marker to better track infection (i.e., RT-qPCR for genome, Western blot for T-antigen).

Fig. 3C: Inclusion of a 2D culture as a control is necessary to interpret the levels from a 3D culture.

Fig. 3E: Similar to Fig. 3B, normalization of the input virus is required to interpret efficiency of infectivity. And again, a better measurement of infection is required.

Fig. 4D: There is no control to indicate that the same copy number or titer of MCPyV was used for infection in the +EV or -EV conditions. It’s unclear whether the additional steps to generate EV-free virus reduced the copy number and thus leads to decreased infection.

Fig. 4E: It’s unclear why the overwhelming majority of detected MCPyV genome is free of capsid and sensitive to DNase. The authors should combine Figs. 4E and 4F; MCPyV DNA within EVs should be resistant to DNase treatment when either proteinase K or chloroform is used independently, but sensitive when combined.

Fig. 4G: Text says that heparin is required for EV-associated MCPyV and naked MCPyV but the figure makes no distinction between the subset of MCPyV used.

Fig. 5A: again, a more robust measurement of infection is required. Additionally, given that infection is blocked by roughly half, writing that “EV membranes can protect virions against neutralizing antibodies” is not valid. This assay should be redone with +EV or EV-free media (with a normalized copy number of MCPyV).

Text at the end of Fig. 5 “These data suggest MCPyV-infected cells could enhance the susceptibility of infection by inducing an immunosuppressive environment that allows pathogens to avoid immune detection and facilitates the spread of infection to neighboring cells.” is not explicitly supported by the data and should be removed or moved to the discussion.

Fig. 6B: The overwhelming factor driving differential expression of downstream Wnt signalling proteins is from the 2D vs. 3D cell culture model. It’s unclear whether MCPyV addition affects this at all. Analysis from the mock 2D vs. 3D models should also be included to determine the relevance of MCPyV infection on these genes.

Fig. 6C+D: 6C finds elevated MMP9 transcripts yet Fig. 6D finds a decrease in active MMP9 protein and a nearly equivalent level of MMP9 pro-form, both of which are in conflict with the elevated transcript finds from Fig. 6C.

Fig. 6E: A more robust measurement of infection is required. Also the text stating “reaffirming that Wnt MMP signaling may play a critical role in regulating the MCPyV lifecycle” is unfounded considering inhibitor 1 had no effect on MCPyV infection. Additionally, it is unclear whether the infection was done in a 2D or 3D model and is not described adequately in the text or figure captions. If this infection was done in the 3D model, it is unclear why an inhibitor against the active MMP9 would decrease infection given that the active form was found to be robustly decreased in the 3D model.

**********

PLOS authors have the option to publish the peer review history of their article (what does this mean?). If published, this will include your full peer review and any attached files.

If you choose “no”, your identity will remain anonymous but your review may still be made public.

**Do you want your identity to be public for this peer review?**  For information about this choice, including consent withdrawal, please see our Privacy Policy.

Reviewer #1: No

Reviewer #2: No

**Figure resubmission:**

-->While revising your submission, we strongly recommend that you use PLOS’s NAAS tool (https://ngplosjournals.pagemajik.ai/artanalysis) to test your figure files. NAAS can convert your figure files to the TIFF file type and meet basic requirements (such as print size, resolution), or provide you with a report on issues that do not meet our requirements and that NAAS cannot fix.

After uploading your figures to PLOS’s NAAS tool - https://ngplosjournals.pagemajik.ai/artanalysis, NAAS will process the files provided and display the results in the "Uploaded Files" section of the page as the processing is complete. If the uploaded figures meet our requirements (or NAAS is able to fix the files to meet our requirements), the figure will be marked as "fixed" above. If NAAS is unable to fix the files, a red "failed" label will appear above. When NAAS has confirmed that the figure files meet our requirements, please download the file via the download option, and include these NAAS processed figure files when submitting your revised manuscript.

**Reproducibility:**

To enhance the reproducibility of your results, we recommend that authors of applicable studies deposit laboratory protocols in protocols.io, where a protocol can be assigned its own identifier (DOI) such that it can be cited independently in the future. Additionally, PLOS ONE offers an option to publish peer-reviewed clinical study protocols. Read more information on sharing protocols at https://plos.org/protocols?utm_medium=editorial-email&utm_source=authorletters&utm_campaign=protocols

# Author comment: Author response to Decision Letter 1

**DOI:** 10.1371/journal.ppat.1014360.r002

28 May 2026

Attachment Submitted filename: Response to Reviewers.docx

# Peer review: Decision Letter 1

**DOI:** 10.1371/journal.ppat.1014360.r003

**Authors:** Blossom Damania, Paul Lambert, Blossom Damania, Paul Lambert

8 Jun 2026

Dear Dr. Kwun,

We are pleased to inform you that your manuscript 'Merkel Cell Polyomavirus Exploits Extracellular Vesicles for Skin Infection and Host Immune Evasion Through Activated Wnt Signaling' has been provisionally accepted for publication in PLOS Pathogens.

Before your manuscript can be formally accepted you will need to complete some formatting changes, which you will receive in a follow up email. A member of our team will be in touch with a set of requests.

Please note that your manuscript will not be scheduled for publication until you have made the required changes, so a swift response is appreciated.

IMPORTANT: The editorial review process is now complete. PLOS will only permit corrections to spelling, formatting or significant scientific errors from this point onwards. Requests for major changes, or any which affect the scientific understanding of your work, will cause delays to the publication date of your manuscript.

Should you, your institution's press office or the journal office choose to press release your paper, you will automatically be opted out of early publication. We ask that you notify us now if you or your institution is planning to press release the article. All press must be co-ordinated with PLOS.

Thank you again for supporting Open Access publishing; we are looking forward to publishing your work in PLOS Pathogens.

Best regards,

Paul F. Lambert

Academic Editor

PLOS Pathogens

Blossom Damania

Section Editor

PLOS Pathogens

Sumita Bhaduri-McIntosh

Editor-in-Chief

PLOS Pathogens

orcid.org/0000-0003-2946-9497

Michael Malim

Editor-in-Chief

PLOS Pathogens

orcid.org/0000-0002-7699-2064

***********************************************************

Reviewer Comments (if any, and for reference):

Reviewer's Responses to Questions

**Part I - Summary**

Please use this section to discuss strengths/weaknesses of study, novelty/significance, general execution and scholarship.

Reviewer #1: The authors satisfied my minor concerns.

Reviewer #2: The present revision has addressed most of our key concerns. Specifically, addressing and controlling for equal input MCPyV in 2D vs. 3D cultures were added. Additionally, more rigorous analyses for infection were added (RT-qPCR and western blotting for T-antigen) to avoid conflation with input virus. Although the molecular mechanisms by which extracellular vesicles and activated Wnt signalling promote increased infection remain unclear, we believe the revision has met our minimum standards for publication.

**********

**Part II – Major Issues: Key Experiments Required for Acceptance**

Please use this section to detail the key new experiments or modifications of existing experiments that should be absolutely required to validate study conclusions.

Generally, there should be no more than 3 such required experiments or major modifications for a "Major Revision" recommendation. If more than 3 experiments are necessary to validate the study conclusions, then you are encouraged to recommend "Reject".

Reviewer #1: (No Response)

Reviewer #2: (No Response)

**********

**Part III – Minor Issues: Editorial and Data Presentation Modifications**

Please use this section for editorial suggestions as well as relatively minor modifications of existing data that would enhance clarity.

Reviewer #1: (No Response)

Reviewer #2: (No Response)

**********

PLOS authors have the option to publish the peer review history of their article (what does this mean?). If published, this will include your full peer review and any attached files.

If you choose “no”, your identity will remain anonymous but your review may still be made public.

**Do you want your identity to be public for this peer review?**  For information about this choice, including consent withdrawal, please see our Privacy Policy.

Reviewer #1: No

Reviewer #2: No

# Editor report: Acceptance letter

**DOI:** 10.1371/journal.ppat.1014360.r004

**Authors:** Blossom Damania, Paul Lambert, Blossom Damania, Paul Lambert

Dear Dr. Kwun,

We are delighted to inform you that your manuscript, "

Merkel Cell Polyomavirus Exploits Extracellular Vesicles for Skin Infection and Host Immune Evasion Through Activated Wnt Signaling," has been formally accepted for publication in PLOS Pathogens.

We have now passed your article onto the PLOS Production Department who will complete the rest of the pre-publication process. All authors will receive a confirmation email upon publication.

The corresponding author will soon be receiving a typeset proof for review, to ensure errors have not been introduced during production. Please review the PDF proof of your manuscript carefully, as this is the last chance to correct any scientific or type-setting errors. Please note that major changes, or those which affect the scientific understanding of the work, will likely cause delays to the publication date of your manuscript. Note: Proofs for Front Matter articles (Pearls, Reviews, Opinions, etc...) are generated on a different schedule and may not be made available as quickly.

Soon after your final files are uploaded, the early version of your manuscript, if you opted to have an early version of your article, will be published online. The date of the early version will be your article's publication date. The final article will be published to the same URL, and all versions of the paper will be accessible to readers.

For Research Articles, you will receive an invoice from PLOS for your publication fee after your manuscript has reached the completed accept phase. If you receive an email requesting payment before acceptance or for any other service, this may be a phishing scheme. Learn how to identify phishing emails and protect your accounts at https://explore.plos.org/phishing.

Thank you again for supporting open-access publishing; we are looking forward to publishing your work in PLOS Pathogens.

Best regards,

Sumita Bhaduri-McIntosh

Editor-in-Chief

PLOS Pathogens

orcid.org/0000-0003-2946-9497

Michael Malim

Editor-in-Chief

PLOS Pathogens

orcid.org/0000-0002-7699-2064
